# A Systematic Review of Trans Fat Reduction Initiatives in the Eastern Mediterranean Region

**DOI:** 10.3389/fnut.2021.771492

**Published:** 2021-11-26

**Authors:** Ayoub Al-Jawaldeh, Mandy Taktouk, Aya Chatila, Sally Naalbandian, Zahra Abdollahi, Buthaina Ajlan, Nawal Al Hamad, Majid M. Alkhalaf, Salima Almamary, Rawan Alobaid, Salah Abdulla Alyafei, Mohammad Hosein Azizi, Nimah M. Baqadir, Rawhieh Barham, Faisal F. Binsunaid, Leila El Ammari, Jalila El Ati, Maha Hoteit, Hanan Massad, Marzeyeh Soleymani Nejad, Lara Nasreddine

**Affiliations:** ^1^Regional Office for the Eastern Mediterranean (EMRO), World Health Organization (WHO), Cairo, Egypt; ^2^Nutrition and Food Sciences Department, Faculty of Agriculture and Food Sciences, American University of Beirut, Beirut, Lebanon; ^3^Science and Agriculture Library, American University of Beirut, Beirut, Lebanon; ^4^Nutrition Department, Ministry of Health and Medical Education, Tehran, Iran; ^5^Nutrition Section, Ministry of Health, Manama, Bahrain; ^6^The Public Authority for Food and Nutrition, Kuwait City, Kuwait; ^7^National Nutrition Committee, Saudi Food and Drug Authority, Riyadh, Saudi Arabia; ^8^Nutrition Department, Ministry of Health, Muscat, Oman; ^9^Senior Regulations and Standards, Saudi Food and Drug Authority, Riyadh, Saudi Arabia; ^10^Health Promotion and Non Communicable Disease (NCD) Division, Public Health Department, Ministry of Public Health, Doha, Qatar; ^11^Food and Beverage Office, Iran Food and Drug Administration (IFDA), Ministry of Health and Education, Tehran, Iran; ^12^Nutrition Department, Ministry of Health, Amman, Jordan; ^13^Healthy Food Department, Saudi Food and Drug Authority, Riyadh, Saudi Arabia; ^14^Nutrition Department, Ministry of Health, Rabat, Morocco; ^15^INNTA (National Institute of Nutrition and Food Technology), SURVEN (Nutrition Surveillance and Epidemiology in Tunisia) Research Laboratory, Tunis, Tunisia; ^16^PHENOL Research Group (Public Health Nutrition Program-Lebanon), Faculty of Public Health, Lebanon University, Beirut, Lebanon; ^17^World Health Organization (WHO), Tehran, Iran

**Keywords:** trans fat, reduction, strategy, implementation, evaluation, Eastern Mediterranean Region

## Abstract

High intakes of trans fatty acids (TFA), particularly industrially-produced TFA, are implicated in the etiology of cardiovascular diseases, which represent the leading cause of mortality in the Eastern Mediterranean Region (EMR). This systematic review aims to document existing national TFA reduction strategies in the EMR, providing an overview of initiatives that are implemented by countries of the region, and tracking progress toward the elimination of industrially-produced TFA. A systematic review of published and gray literature was conducted using a predefined search strategy. A total of 136 peer-reviewed articles, gray literature documents, websites and references from country contacts were obtained, up until 2 August 2021. Randomized-control trials, case-control studies, and studies targeting unhealthy population groups were excluded. Only articles published after 1995, in English, Arabic or French, were included. Key characteristics of strategies were extracted and classified according to a pre-developed framework, which includes TFA intake assessment; determination of TFA levels in foods; strategic approach; implementation strategies (TFA bans/limits; consumer education, labeling, interventions in public institution settings, taxation), as well as monitoring and evaluation of program impact. Thirteen out of the 22 countries of the EMR (59%) have estimated TFA intake levels, 9 have determined TFA levels in foods (41%), and 14 (63.6%) have national TFA reduction initiatives. These initiatives were mainly led by governments, or by national multi-sectoral committees. The most common TFA reduction initiatives were based on TFA limits or bans (14/14 countries), with a mandatory approach being adopted by 8 countries (Bahrain, Iran, Jordan, KSA, Kuwait, Morocco, Oman and Palestine). Complementary approaches were implemented in several countries, including consumer education (10/14), food labeling (9/14) and interventions in specific settings (7/14). Monitoring activities were conducted by few countries (5/14), and impact evaluations were identified in only Iran and the UAE. The robustness of the studies, in terms of methodology and quality of assessment, as well as the lack of sufficient data in the EMR, remain a limitation that needs to be highlighted. Further action is needed to initiate TFA reduction programs in countries that are lagging behind, and to ensure rigorous implementation and evaluation of ongoing programs.

## Introduction

Cardiovascular diseases (CVD) represent the leading cause of mortality worldwide, causing ~17.9 million deaths each year and contributing to 31% of global deaths (1). High intake of trans fatty acids (TFA), particularly industrially produced TFA, were implicated in the etiology of CVD (2). Although available data on TFA intake globally is rather limited, it was recently reported that the 2017 global market volume of partially hydrogenated oils (PHOs), which is the major source of industrially produced TFA in food, was around 13.6 million tons (3). Wang et al. showed that compared to an optimal TFA intake of 0.5% of energy intake (%EI), excess TFA consumption was estimated to cause 537,200 coronary heart disease (CHD) deaths per year worldwide in 2010, representing 7.7% of global CHD mortality (4).

This is consistent with the unique cardiometabolic imprint of industrial TFA on both lipid and non-lipid pathways (4, 5). Physiologically, TFAs impact the lipid profile, raising the levels of the atherogenic low-density lipoprotein (LDL) cholesterol while also decreasing the levels of the cardioprotective high-density lipoprotein (HDL) cholesterol (2, 6). Randomized controlled trials have also shown that high TFA consumption produces adverse cardiovascular effects via pathways linked to the insulin resistance syndrome (5). A reduction in the population's intake of TFA has therefore been acknowledged as one of the policy priorities adopted by the World Health Organization (WHO) (5). One of the core indicators of the WHO global framework for monitoring non-communicable diseases (NCDs) by 2025, is the “adoption of national policies that virtually eliminate partially hydrogenated vegetable oils in the food supply and replace [them] with polyunsaturated fatty acids” (7). The elimination of industrially produced TFAs is in fact a relatively straightforward, low-cost and effective policy measure that is within reach of most countries, while carrying substantial long-term health benefits (3, 8, 9). However, most countries worldwide have yet to develop and implement strong policy measures to limit TFA intakes, and TFAs continue to be highly consumed around the world (10). In May 2018, the WHO has advocated for the global elimination of industrially produced TFA by 2023 and released the REPLACE action framework, with the aim of decreasing CVD-related mortality (3, 11). The REPLACE action framework consists of a roadmap for countries to implement immediate, complete and sustained elimination of industrial TFA from the food supply (12).

In the Eastern Mediterranean Region (EMR), a region that harbors a high burden of CVD, and where the consumption of TFA is high (13, 14), the WHO Regional Office for the Eastern Mediterranean, in collaboration with WHO Headquarters and Resolve to Save Lives, has been providing technical assistance to support country-level policy development and implementation. The WHO EMR regional nutrition strategy 2020–2030, has included specific objectives related to the virtual elimination of industrially-produced TFA from the food supply (15), and recommended a number of priority actions that will assist Member States in reaching these objectives (15). However, to date, there has been no comprehensive appraisal of the government-initiated public health strategies that are being adopted by countries of the region in order to eliminate TFA from the food supply and decrease the population's TFA intake. It is in this context that we conducted this systematic review, with the aim of identifying and documenting existing national TFA reduction strategies, providing an overview of initiatives that are implemented by countries of the EMR to reduce TFA intakes, and tracking progress toward the goal of TFA elimination by 2023.

## Materials and Methods

**Figure 1:**
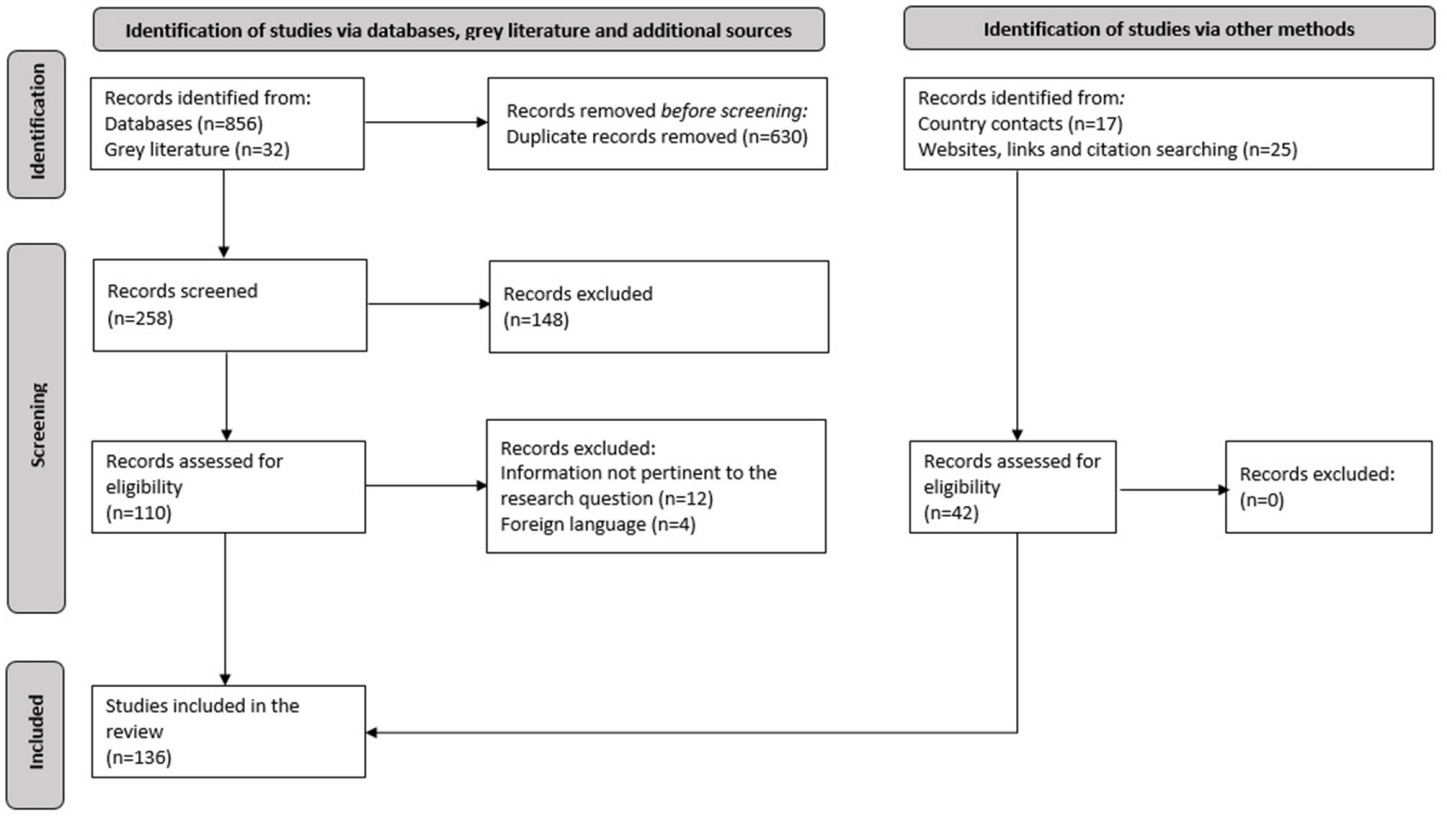
Search and identification process of potential references from the literature.

The methodology and search strategy adopted in this study were similar to the approach described by Santos et al. (16) in their systematic review of salt reduction strategies. Accordingly, data related to TFA reduction initiatives were obtained through a series of steps, allowing for maximum coverage of the EMR and its 22 countries (17). This comprised a search of peer-reviewed and gray literature published up to 15 April 2021, in addition to seeking supplementary information by directly contacting program country leaders or focal points (Figure 1).

### Search Strategy

The present systematic review conforms to the Preferred Reporting Items for Systematic Reviews and Meta-Analyses (PRISMA) statement. A total of 12 electronic databases were searched between 17 February 2021 and 15 April 2021. These databases included: CAB Direct (18), Directory of Open Access Journals (19), Google (20), Ovid (21), National Library of Medicine (22), Elsevier (23), Clarivate (24), EBSCO (25), Al Manhal (26), EBSCO (27), E-Marefa (28) and Ministry of Higher Education and Scientific Research of Iraq (29); the last four being databases specific to the Arab region. In addition to using controlled vocabulary (MeSH in PubMed and MEDLINE), a comprehensive list of search terms was used in the title/abstract/keyword fields to cover the four concepts (1) TFA, (2) reduction OR intake, (3) policy, and (4) EMR countries. MEDLINE (Ovid) was searched first using a combination of MeSH terms and keywords. The search strategy was mapped to PubMed after making the necessary changes. To search the remaining databases, MeSH terms were searched as keywords in title-abstract-keyword fields whenever applicable. Google Scholar was searched using title field only. Appendix A shows the detailed list of search terms used in PubMed. Moreover, an example of a database search on PubMed is shown in Supplementary Table 1. The search excluded any material published before 1995. Only English, Arabic and French languages were considered. Newly published articles after the initial search were identified by email alerts (up until 2 August 2021).

A search of the gray literature was also performed, using OpenGrey (30), Google (31), World Healh Organization (32), World Health Organization Regional Office for the Eastern Mediterranean (33) and governmental websites (e.g., Ministries of Health). The search was limited to materials published post 1995 in English, Arabic and French languages only.

EndNote X9 (Version 18.0.0.10063) was used for the export of all identified articles after conducting the search on online databases and gray literature. Two independent researchers (MT and AC) screened the titles, abstracts and full text articles of the potentially relevant articles, according to the inclusion and exclusion criteria mentioned in the below section. The two researchers discussed and resolved the minor discrepancies that resulted from the two screening stages.

### Inclusion and Exclusion Criteria

Articles were included in this review if they provided information on TFA baseline assessment [intake; levels in foods; knowledge, attitudes and behavior (KAB)], or the development, implementation, monitoring or evaluation of national TFA reduction initiatives at the national level. National TFA reduction initiatives were defined as having a governmental entity involved (12, 34, 35), in addition to one or more of the following components: (1) a national action plan to reduce the population TFA intake (12); (2) a program of work on TFA replacement (e.g., banning the use of partially hydrogenated oils; setting limits for industrial TFA in food products) (36); (3) consumers' education programs or awareness campaigns with the aim of improving KAB toward TFA (12); (4) labeling schemes specific to TFA or mandatory declaration of TFA on nutrition labels (12, 36); (5) taxation policies targeting high-TFA foods, or unhealthy foods defined by their high TFA content (12); and (6) TFA reduction initiatives in specific settings (schools, hospitals, workplaces) (12).

Articles based on randomized-control trials or case-control studies, as well as those targeting unhealthy individuals or specific populations (pregnant women, individuals on therapeutic diets etc.) were excluded. Individual articles were also excluded if they were published before 1995, or in any language besides English, Arabic, or French.

### Data Extraction

Data extraction was conducted independently by two researchers (MT and AC), and then a third researcher (LN) reviewed the data for accuracy. The researchers discussed the few discrepancies until reaching consensus. For each national TFA reduction initiative, the key characteristics were entered into a database constructed by the researchers, and examined in relation to baseline assessments (population TFA intake; TFA levels in food products, KAB related to TFA), leadership and strategic approach, implementation strategies (TFA bans or limits; consumer education, food labeling, interventions in public institution settings, taxation), monitoring (population intake, levels in food products, KAB), and evaluation of program impact (12, 34, 36, 37).

### Seeking Supplementary Information

To seek supplementary information regarding national TFA reduction initiatives, a questionnaire was developed by the research team based on relevant literature (6, 37, 38) (Supplementary Questionnaires). After its development, the questionnaire was reviewed by a nutritionist and a public health professional for content validity. The questionnaire was then sent to country experts or program leaders in various countries of the EMR to verify and obtain supplementary country-specific data. Country experts or program leaders were invited to fill the questionnaire and/or pass on the questionnaire to their contacts to obtain additional information and deliver the needed details. The database was then updated accordingly with the additional obtained data.

### Analysis

For each identified national TFA reduction strategy, the core characteristics were entered into the database, according to the developed framework that includes baseline assessments; leadership/strategic approach; the different types of implementation strategies; monitoring data; and evaluation of program impact. Countries were then categorized as “having a developed strategy” for TFA elimination/reduction, “having a planned strategy” or “having no strategy.” Strategies were considered to be “planned” if the TFA reduction initiatives were still being developed or if an action plan had been already developed but without evidence of implementation. A quantitative evaluation of the proportion of countries reporting on each core characteristic was performed, and expressed as percentages.

## Results

### Search Results

A total of 136 peer-reviewed articles, gray literature documents, websites and references from country contacts were obtained from the literature search; 94 were peer-reviewed articles of potential relevance and 42 were additional sources obtained from country contacts via the completed questionnaires, links, webpages, and references from within the included studies (Figure 1).

### Assessment of TFA Intake

Out of the 22 countries of the EMR, thirteen (59%) did not have any population estimate of TFA intakes (with the exception of estimates based on Bayesian modeling and which were not considered in this review) (10). In contrast, nine countries (41%), including Egypt, Iran, Jordan, Lebanon, Morocco, Pakistan, Sudan, Tunisia, and the UAE, have estimated population TFA intakes (Supplementary Table 2). Some of the available studies estimated TFA intakes at the national level for specific population groups (e.g., adults) or the entire population (per capita), while others reported the intakes within specific regions of the countries (4, 12, 14, 39–51). Except for Jordan, where TFA intake assessment was based on a household budget survey, all the other countries had evaluated TFA intakes based on dietary assessment methodologies (e.g., 24-h recalls, food frequency questionnaires, diet history questionnaires) (Supplementary Table 2). Total diet studies investigating dietary exposure to TFA are lacking in the region.

**Figure 2:**
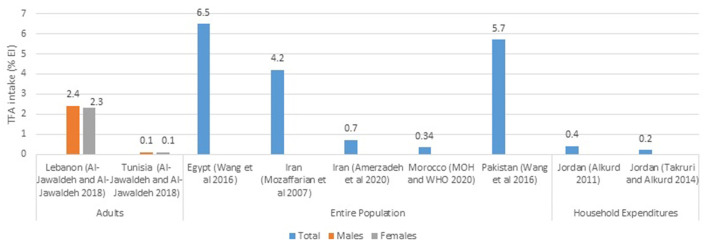
TFA intake estimates (%EI) based on national studies in countries of the EMR. EI, energy intake; EMR, Eastern Mediterranean Region; MOH, Ministry of health; TFA, trans fatty acids; WHO, World Health Organization. References: For Adults: Lebanon and Tunisia (dietary assessment): Al-Jawaldeh and Al-Jawaldeh (14). For Entire Population: Egypt and Pakistan (dietary assessment): Wang et al. (4); Iran (dietary assessment): Mozaffarian et al. (39); Iran (consumption data): Amerzadeh and Takian (41); Morocco (consumption data): Ministry of Health-Morocco and World Health Organization (49). For Household Expenditures: Jordan: Alkurd (44) and Takruri and Alkurd (45).

**Figure 3:**
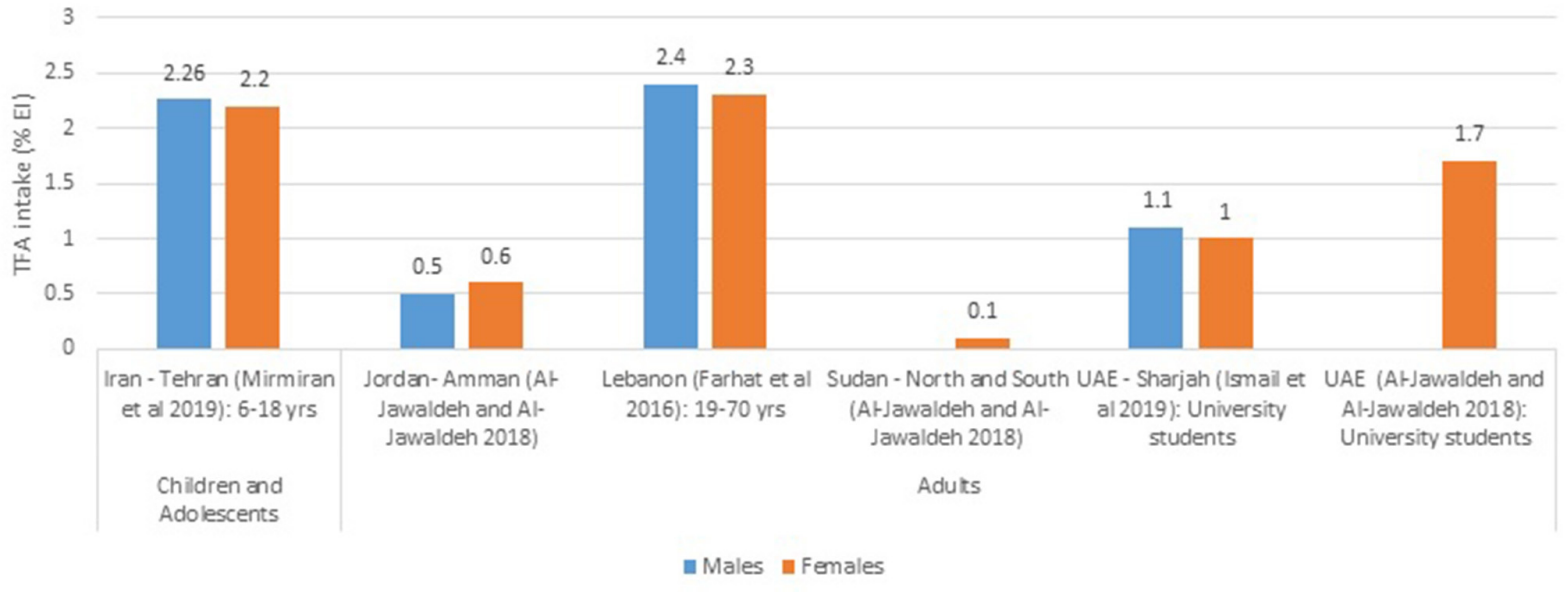
TFA intake estimates (%EI) based on regional studies in countries of the EMR. EI, energy intake; EMR, Eastern Mediterranean Region; MOH, Ministry of health; TFA, trans fatty acids; WHO, World Health Organization. References: For Children and Adolescents: Iran (dietary assessment): Mirmiran et al. (43). For Adults: Jordan and Sudan (dietary assessment): Al-Jawaldeh and Al-Jawaldeh (14); Lebanon (dietary assessment): Farhat et al. (48); UAE (dietary assessment): Ismail et al. (51) and Al-Jawaldeh and Al-Jawaldeh (14).

The available estimates of TFA intakes from various countries are presented in Figure 2, which focuses on national studies, and Figure 3, which illustrates data stemming from regional studies within countries. Based on national per capita estimates of TFA intakes (entire population), the highest level was observed in Egypt (6.5% EI), while the lowest was reported from Morocco (0.34% EI) (Figure 2). As for regional studies conducted amongst adults (Figure 3), the highest levels were observed in Lebanon (2.3–2.4% EI), while the lowest were noted among females in Sudan (0.1% EI) (Figure 3). Few countries have evaluated TFA intake among children and adolescents. In Iran this was reported at 2.2% EI in girls and around 2.3% in boys (Figure 3). In Jordan, TFA intake was estimated to range between 0.8 and 1.3 g/day among 6–18 year old children (46), and in Lebanon, average intake of TFA was reported at 0.16 g/day in 5–10 year old children (47). The contributions of these estimates to energy intake were not reported.

### Assessment of TFA Levels in Food and TFA-Related KAB

Several countries in the EMR (9/22 countries; 41%), including Egypt, Iran, Jordan, Kingdom of Saudi Arabia (KSA), Kuwait, Lebanon, Morocco, Pakistan, and Tunisia, have evaluated TFA levels in local foods and commodities (Supplementary Table 3) (14, 39, 49, 52–94). The majority of available TFA content data were derived based on chemical analysis of food products (39, 49, 52–56, 58, 60–70, 72–74, 77, 79–97), while very few studies have collected TFA content data based on food labels and packages (55, 75, 76). Most of the available studies have reported very high levels in fast foods (fried meats, sausages, French fries), pastries, potato chips, biscuits, wafers, cakes, donuts, chocolates, traditional sweets, as well as dairy products (milk, cream, cheeses). Interestingly, available data indicate that the levels of TFA in imported foods were higher as compared to locally produced items. For instance, Mashai et al. (72) showed that the levels of TFA in five imported popcorn items ranged from 2% to over 40% as compared to 1.26% in a locally produced popcorn. In addition, in KSA, Jradi et al. (76) have randomly collected 1,153 foods from fourteen stores in the Saudi market. Results showed that from the 228 products that had hydrogenated fat, 67.5% were imported, while 32.5% were locally produced (76).

Iran was the only country that showed decreasing TFA levels in vegetable oil, margarines and shortenings over time (Supplementary Table 3). Acknowledging that different countries/studies may have used various methods for the chemical determination of TFA levels in foods, the WHO has spearheaded the development of a standardized chemical analysis protocol for TFA assessment (98). This has been already implemented in Egypt and Jordan (94, 97), as well as Lebanon and Morocco (data not published yet) (Supplementary Table 3).

As for baseline data on KAB, this was collected in eight EMR countries (32%), including Iran, Iraq, Jordan, KSA, Oman, Pakistan and the UAE. Most of the KAB surveys included questions relating to (1) knowledge of TFA food sources and content, familiarity with the maximum daily allowance of TFA and the adverse health effects of high TFA intakes; and (2) consumers' behavior such as the use of partially hydrogenated oils and fats when cooking, or the consumption of high TFA food products (Supplementary Table 4) (75, 99–107). No studies assessed the attitudes of consumers toward TFA.

### Countries With National TFA Reduction Initiatives

As shown in Table 1, national TFA reduction initiatives were identified in 14 out of the 22 countries of the EMR (63.6%). These countries include Bahrain, Egypt, Iran, Iraq, Jordan, KSA, Kuwait, Morocco, Oman, Pakistan, Palestine, Qatar, Tunisia and the UAE. GINA database has included a TFA reduction policy for Lebanon (151). More specifically, it referred to limiting the import, fabrication and marketing of high TFA products−2014. However, the GINA database has also specified that this initiative was not adopted in the country (151). Accordingly, and given that no other TFA reduction initiatives were identified during our systematic search, Lebanon was not considered as having national TFA reduction initiatives in this review. Similarly, it was reported that Afghanistan and Syria are preparing broad national nutrition or food policies, which will include TFA reduction (108), and that legislation/standards related to TFA limits in fats and oils and/or the use of PHOs are being developed or updated in Sudan (108, 109). However, no further details were found, and accordingly Afghanistan, Sudan and Syria were not considered as having TFA reduction initiatives in this review.

**Table 1:** TFA reduction implementation strategies in countries of the EMR.

**Country**	**TFA bans or limits**	**Consumer Education/Behavior Change**	**Labeling**	**Work in Specific Settings**
Bahrain	Name of initiative: A standard relating to TFA elimination. Decision of the Ministry of Industry, Trade and Tourism No. (16) of 2016 regarding Policy–Adopting the GSO technical regulations for food products as national technical regulations Year: 2016 Leadership: Led by the government (MOH-Public Health Directorate, Ministry of Industry, Trade and Tourism) and GSO Approach and target: Mandatory: for all food, oils and snack categories (e.g., maximum TFA content in foods and snacks or zero TFA in foods and snacks). The standard also specifies a maximum level of 2% in vegetable oils and soft spreadable margarines and 5% in all other foods, including ingredients sold to restaurants (bakery products, fast food, restaurant frying oil, biscuits and cakes, and salty snacks) (4, 108–111)	Name of initiative: Media campaign to raise the awareness of community on trans fat and workshops targeting the food importers and the food industry Year: 2017 Leadership: Led by the government (MOH and Ministry of Industry and Trade) Approach: Social marketing (e.g., campaigns); Media (TV and radio); Booklets; Workshops targeting food importers and the food industry Information provided by the NFP	Name of initiative: GSO 2483/2015 (E) Year: 2016 Leadership: Led by GSO Approach: Mandatory: provides standards which mandate the nutritional labeling of fat, including TFAs, as g/100 g and %DV (50, 111, 112), requiring declaration of TFA as part of nutrition labels for products containing 0.5/100 g or more and regulating “trans-fat free” claims (108, 111)	Name of initiative: Eliminating TFA from school canteens through the adoption of a prohibited food list and by modification of food preparation/cooking methods Year: 2017 Leadership: Led by the government (MOH and Ministry of Education) Approach: Procurement policy, workshops; the healthy food list for school canteen and the required standards for preparation Setting: Schools Information provided by the NFP
Egypt	Name of initiative: NA Year: NA Leadership: Led by the government (MOH, with the participation of all sectors) Approach: A roadmap for action on industrial TFA elimination has been drafted. Some factories are reported to have started using esterification technology, on a voluntary basis, to eliminate the hydrogenation process (108, 109)	–	–	–
Iran	Name of initiative: TFA reduction in edible oils and foods Year: 2008–2016 Leadership: Led by the National Standard Organization and High Council of Health and Food Security Approach and targets: Mandatory. In 2008, the National Standard Organization mandated the revision of standard NO.9131, so that TFAs contents of edible oils (both imported and local) are limited to 5% In 2014, the High Council of Health and Food Security approved to revise the standards of TFAs to <2% (14, 41, 50, 113–115). The permitted levels of frying oil and mixed liquid oils were reduced to <2% by 2015 (108, 116); TFA levels in table margarine from 10 to 2%, in spread margarine and minarine from 5 to 2%, after 2015 (117, 118). As of 2016, the 2% limit in fats and oils became effective (4, 108, 110, 119). For some other products, the upper limit is 5% (e.g., shortening for bakery products) (57, 108, 110, 116), while for others such as biscuits it should be under 2% of extracted fat from the biscuit (57) Initiative: Reduction of TFA in edible oils Year: 2005 Leadership: Led by an executive committee composed of members from the MOHME, Ministry of Industry, Ministry of Agriculture, Ministry of Commerce and the National Standard Organization Approach and targets: Ministry of Commerce was forced to gradually replace the hydrogenated oils (the subsidized ones) by non-hydrogenated (specifically olive oil) and liquid frying oils (14, 50, 113). The standards for frying oil and mixed liquid oils were revised in 2005 to reduce the maximum level of TFA (to <10%, down from over 20%) (120)	Name of initiative: National plan for the reduction of TFA and SFA in the Iranian diet Year: NA Leadership: Led by the government (MOHME) Approach: Public education campaign with emphasis on increasing the public's knowledge about the adverse health effects of TFA in processed food and edible oils. Mass media, magazines and newspapers were used for campaign promotion to increase people's awareness (105, 121) Name of initiative: Educational campaigns for increasing awareness on salt and sugar, total fat and TFA reduction Year: Annually since 2010 Leadership: Led by the government Approach: Social marketing (e.g. campaigns), TV advertising and events. Information provided by the NFP	Name of initiative: NA Year: 2008 Leadership: Led by the government (MOHME) and the National Standard Organization Approach: Reviewing packaging standards and mandating manufacturers and importers to affix labels to all food products, especially edible oils (14, 113) Name of initiative: Food labeling modification through designing Traffic Light Symbol Year: 2016 Leadership: Led by the Food and Drug Organization and MOHME (government and industry led) Approach: Mandatory: percentage of daily intake. Information provided by the NFP	Name of initiative: NA Year: NA Leadership: NA Approach: Educational work on TFA has been done such as inclusion of TFA related information in school books Setting: Schools Information provided by the NFP
Iraq	Name of initiative: NA Year: NA Leadership: NA Approach: Subsidy on hydrogenated ghee/shortening removed and replaced by other types of oil (14, 50, 122) No further details	–	–	–
Jordan	Name of initivative: Withdraw the Jordanian Technical Base; Reformulation of specifications and regulations (1520/200l and 1605/2004) to eliminate TFA in cheese products Year: 2016 Leadership: Led by the government (MOH and JSMO) Approach: Mandatory technical specifications and regulations were developed to reduce TFA in processed cheese (adopted by JSMO). Moreover, MOH, in collaboration with JSMO, banned the use of PHOs in dairy products (local and imported) in 2016. The decree states that only animal fat occurring naturally in dairy products be permitted in milk and cheese (50, 123, 124)	Name of initiative: Low salt, low sugar, low saturated and trans fat consumption guideline for health care providers for training of trainers (TOT) and pamphlet for consumers Year: 2015 Leadership: Led by the government (MOH) Approach: Awareness and education (125) Name of initiative: Guideline for low consumption of salt, sugar, saturated fat and trans fat Year: 2020 Leadership: Led by the government Approach: Inclusion in FBDG (126)	Name of initiative: National Strategy and Plan of Action Against Diabetes, Hypertension, Dyslipidemia and Obesity Year: 2015 Leadership: Led by the government Approach: Mandatory: labeling of food products with TFA (not adopted yet) (4, 127)	Name of initiative: Reduce trans fat in canteens of public hospitals and royal medical services hospitals for inpatients and employees Year: 2019 Leadership: Led by a multi-sectoral committee and WHO Approach: Banning TFA in public sector hospital food, and increasing portions of olive and sesame oil in foods Setting: Public sector hospitals and royal medical services (108)
KSA	Name of initiative: Healthy Food Strategy Year: 2018 Leadership: Led by the government (SFDA) Approach and targets: In 2017, mandatory for all food and snack categories. The maximum limit of TFA in vegetables oils and margarine shall be 2% and the maximum limit in other foods (such as fast foods, restaurant frying oils, biscuits and cakes) shall be 5% of the total content of fats (4, 108, 110, 111, 128–131). In 2018, voluntary pledge to avoid the use of PHOs in food products with major food producers around the globe (112) In 2020, and as part of Healthy Food Strategy, KSA banned the use of PHOs in food products (0 g); it was voluntary until January 2020 when it became mandatory (108, 110, 128, 129, 132)	Name of initiative: The Healthy Food Guide for the health practitioner Year: 2020 Leadership: Led by the government (MOH) Approach: Designed to educate health care workers about the different macronutrient distribution (including TFA), their sources; provides information on the content of a full day meal, and fosters proper reading of the labels (133) Name of initiative: Healthy Food Strategy Year: 2018 Leadership: Led by the government (SFDA) Approach: Social media awareness campaigns, workshops and events (131)	Name of initiative: Reduction of TFA, and Healthy Food Strategy SFDA.FD 2483/2017 (E) SFDA.FD 2233/2018 (E) Year: 2017–2020 Leadership: Led by the government (SFDA) Approach: Mandatory: nutrition labels should include the amount of TFA (g/100 g), and the declaration of fat/oil names among the ingredients list (50, 111, 112, 128). Declaration of TFA as part of nutrition labels for food products containing 0.5 g/100 g or more and regulating “trans-fat free” claims (108, 110, 111, 128) Name of initiative: The Nutritional Label Guide Year: 2020 Leadership: Led by the government (SFDA) Approach: Mandatory: the fat value indicates the total fat: SFA in red, TFA in green, cholesterol in blue. Mandatory indication “Make sure your food is free of trans fats” (134)	Name of initiative: Guidelines for the government nutritional subsistence purchase contract Year: 2019 Leadership: Led by the government (SFDA) Approach: Provides UFAs (canola and corn oil instead of SFAs) and bans the use of PHO in foods Setting: Government subsistence nutritional purchase contract in hospitals, universities, military or social places (not adopted) (135) Name of initiative: Healthy Food Strategy Year: 2015 Leadership: Led by the government (SFDA) Approach: Education Setting: Schools, hospitals and the workplace (131)
Kuwait	Name of initiative: Standards relating to TFA elimination Year: 2017 Leadership: Led by GSO and the Public FNA, the Kuwait focal point and official representative at GSO and the Ministry of Industry and Trade Approach and targets: Mandatory: specifies a maximum level of 2% in vegetable oils and soft spreadable margarines and 5% in all other foods, including ingredients sold to restaurants (4, 108, 111, 136, 137)	Name of initiative: NA Year: 2020 but stopped due to COVID-19 pandemic (Planned to resume after Ramadan 2021) Leadership: Led by the Community Nutrition Sector in coordination with the Department of Nutrition Promotion and Education Approach: Health and Nutrition Education targeting: Manufacturers: raising awareness among manufacturers and suppliers of the adverse health effects of TFAs and finding alternatives Consumers: raising awareness through educational campaigns for all age groups to explain the adverse health effects of TFA; to read the nutrition label and identify the products that should be limited/avoided Information provided by the NFP	Name of initiative: GSO 2,483/2015 (E) Year: 2016 Leadership: Led by the Public FNA, the Kuwait focal point and official representative at GSO, Kuwaiti Ministerial decision—Ministry of Industry and Trade Approach: Mandatory: adoption of GSO standard and regulation pertinent to TFA (GSO 2483); this standard applies to the maximum amount allowed for TFA and declaring the TFA content on the nutrition label per serving, as g/100 g and %DV—back-of-pack labeling (50, 108, 111, 112, 137), while requiring declaration of TFA as part of nutrition labels for products containing 0.5 g/100 g or more and regulating “trans-fat free” claims (108, 111). A new TFA regulation will be implemented from 1 January 2021 (108, 137) Name of initiative: Kuwait Action Plan for SFA Intake Reduction and TFA Elimination Year: 2012 Leadership: Led by the FNA and the MOH Approach: Review GSO proposal for SFA and TFA standards (138)	Name of initiative: The role of the Authority of Food and Nutrition, Ministry of Health in the control and prevention of NCDs in Kuwait Year: 2021 Leadership: Led by the FNA on behalf of the Kuwait MOH, in cooperation with the PHFS Approach: Apply the traffic light system on food items sold in governmental hospital cafeterias and canteens (139) Name of initiative: Nutrition-Friendly Schools Initiative Year: NA Leadership: Led by the government (MOH) Approach: Raising nutrition and health awareness through continuous education program Setting: Schools (136, 140) Name of initiative: Kuwait Action Plan for SFA Intake Reduction and TFA Elimination Year: 2012 Leadership: Led by Kuwait's Salt and Fat Intake Reduction Task Force and the Public FNA Approach: Banning the use of TFA in governmental institutions (MOH hospitals, Ministry of Defense, Ministry of Interior, public authorities, schools and universities) (not adopted) (138)
Morocco	Name of initiative: Reduction of TFA content in food products Year: 2020 Leadership: Led by the government Approach and targets: Voluntary in general, however, for certain items such as oils, fats and snacks it is mandatory. Setting the limit for TFA content in oils and fats (such as margarines, spreads, vegetable oils, restaurant frying oils) at 2 g/100 g of product; and at 5 g/100 g of product in all other foods (such as salty snacks, biscuits and cakes) (11, 49). Moreover, there has been a decree proposal by the Ministry of Agriculture, in 2021, to ban the partial hydrogenation of vegetable oils (Planned).= Information provided by the NFP	–	–	–
Oman	Name of initiative: Ministerial decision No. 2019/95 Omani standard for bread Year: 2019 Leadership: Led by the government (MOCI) Approach: Mandatory: bread benchmark for TFA should not exceed that set by GSO (141) Name of initiative: A standard relating to TFA elimination Year: Planned for initiation Leadership: Led by GSO the Ministry of Commerce Approach and targets: Mandatory: specifies a maximum level of 2% in vegetable oils and soft spreadable margarines and 5% in all other foods, including ingredients sold to restaurants (4, 108, 111) Name of initiative: Summary of proposed implementation mechanisms to reduce saturated and trans fat consumption 2016–2020 Year: 2016 Leadership: NA Approach: Proposed mechanisms to reduce the use of TFAs in industries, restaurants (not adopted) (142) Name of initiative: National plan for the prevention and control of chronic NCDs 2016–2025 Year: 2016 Leadership: Led by the government (MOH) Approach: Gradually shift from TFAs toward healthier types of fats to reach a target of 100% by 2025; taxation on the use of hydrogenated oils to reach a target of 50% by 2025 (not adopted) (143)	Name of initiative: Healthy Nutrition Campaign Year: 2021–2022 Leadership: Led by the government (MOH) and NGO Approach: Social marketing, TV advertising, and events (Planned) Information provided by the NFP Name of initiative: Summary of proposed implementation mechanisms to reduce saturated and trans fat consumption 2016–2020 Year: 2016 Leadership: NA Approach: Proposed mechanisms to spread awareness among consumers regarding impact of TFAs on health (142)	Name of initiative: GSO 2483/2015 (E) Year: 2016 Leadership: Led by GSO Approach: Mandatory: provides standards which mandate the nutritional labeling of fat, including TFAs, as g/100 g and %DV (50, 111, 112, 142), requiring declaration of TFA as part of nutrition labels for products containing 0.5 g/100 g or more and regulating “trans-fat free” claims (108, 111, 142). Voluntary: GSO standard for labeling of prepackaged food stuffs; include the percent daily intake (144). Name of initiative: NA Year: 2014 Leadership: Led by the government Approach: Require food importers to have all imported foods certified as industrial TFA free (not adopted) (145)	Name of initiative: National plan for the prevention and control of chronic NCD 2016-2025 Year: 2016 Leadership: Led by MOH Approach: Limit the availability of high TFA items Setting: Schools (not adopted) (143)
Pakistan	Name of initiative: Punjab Pure Food Regulations Year: 2018 Leadership: Led by Punjab Food Authority Approach and targets: TFA content in Vanaspati, table margarine, industrial margarine, margarine spread, spread and shortening shall not have more than 0.5% TFA. After July 2020, there shall be complete ban on any form of Vanaspati. For frying oils and fats, TFA should not be more than 5% (82, 105) Name of initiative: Punjab Pure Food Rules Year: 2011 Leadership: Led by the government and KP Food Safety and Halal Food Authority Approach and targets: The content of TFAs shall not exceed 3% of total fatty acids provided 100% milk fat is used in the formula (82, 105, 146); the use of commercially hydrogenated oils is prohibited; plant oils and fats intended to be used in follow-up formula should be virtually TFA-free and the maximum allowance level of TFA shall be proportionately decreased with increasing level of plant oils and fats in the formula (146) Name of initiative: PS 221 (Pakistan Standard Specifications for Vanaspati) Year: NA Leadership: Led by PSQCA Approach: Regulate production and import of vanaspati ghee, margarine, butter and a number of oil products under a list of compulsory items Target: TFA limit (<5%). It has also been proposed to bring TFA level at par to the guidelines of WHO by year 2023 in this mandatory standard (82)	–	Name of initiative: Punjab Pure Food Regulations and KP Food Regulations Year: 2018 Leadership: Led by Punjab Food Authority and KP Food Safety and Halal Food Authority Approach: TFA percentage of Vanaspati shall be mentioned on the label All cream analogs shall mention TFA contents on the label. The label shall also mention source of vegetable oil(s) used in their descending order Margarine shall be clearly defined on the label in Urdu “ye makhan nahi hai” (“this is not butter”). This label shall be 15% of the total package area and it shall be mentioned on both sides of the label, in two colors only. The TFA percentage must be mentioned on the label Dried ice cream mix/dried frozen dessert/confectionary should also declare % TFA per serving (82, 105)	–
Palestine	Name of initiative: Amendment of the mandatory technical instructions 2011–2032 by adding vitamins, minerals and other specific substances to food Year: 2021 Leadership: Led by the government (MOH and Ministry of National Economy) Approach and targets: Mandatory: a maximum of 2 g TFA/100 g in prepared foods (147)	Name of initiative: National Nutrition Policy, Strategies and Action Plan 2017–2022 Leadership: Led by the government (MOH) Year: 2017–2022 Approach: Conducting awareness campaigns regarding the importance of reducing TFA intake (148)	–	–
Qatar	Name of initiative: Initiative to reduce fat, sugar and salt consumption in Qatar Year: 2019 Leadership: Led by the government (MOPH) Approach: Voluntary: targets for TFA levels in foods and snacks (Planned). Information provided by the NFP Name of initiative: A standard relating to TFA elimination Year: 2020 (submitted for endorsement) Leadership: Led by government (MOPH and Ministry of Municipality and Environment) and GSO Approach and targets: Voluntary: specifies a maximum level of 2% in vegetable oils and soft spreadable margarines and 5% in all other foods, including ingredients sold to restaurants (4, 108, 111). In 2020 the GSO standard 2,483 had been submitted to the cabinet for endorsement (108)	Name of initiative: Initiative to reduce fat, sugar and salt consumption in Qatar Year: 2019 Leadership: Led by the government (MOPH) Approach: Social marketing (e.g. campaigns), TV advertising, events, TV and radio interviews, inclusion in FBDG Information provided by the NFP	Name of initiative: GSO 2483/2015 (E) Year: 2016 Leadership: Led by GSO Approach: Mandatory: provides standards which mandate the nutritional labeling of fat, including TFAs, as g/100 g and %DV (50, 111, 112), requiring declaration of TFA as part of nutrition labels for products containing 0.5 g/100 g or more and regulating “trans-fat free” claims (108, 111)	Name of initiative: Food & Beverage Guidelines; School Canteen Guidelines; Educational sessions in schools and workplaces Year: Ongoing Leadership: Led by the government (MOPH) Approach: Education, procurement policy (planned), voluntary guidelines. School Canteen Guidelines are mandatory in governmental schools Setting: Schools, hospitals, workplace Information provided by the NFP
Tunisia	Name of initiative: NA Year: 2015 Leadership: Led by the food industry (one manufacturer) Approach: The manufacturer launched a margarine product without TFAs after adapting new food processing technology (14, 50) Tunisia is advocating for the implementation of legislation to ban the production/importation of industrial TFA (130)	Name of initiative: Strategy for the Prevention and Fight against Obesity Year: 2012 Leadership: Led by the government (MOH) Approach: Reducing TFA intake and helping consumers make healthier choices that provide less TFA (149)	–	–
UAE	Name of initiative: National Action Plan in Nutrition Year: 2017 Leadership: Led by GSO; MOHP of the UAE Approach and targets: Reformulate food products; Replace TFAs with UFAs; Reduction of TFA to maximum 2 g of the total fat in vegetable oils, and soft spreadable margarine, and TFA content for other foods to <5% of the total fat content including ingredients sold to restaurants (GCC legislation on TFA approved). MOHAP Task Force on Reduction of TFA in the UAE established) (4, 108, 111, 150)	Name of initiative: National Action Plan in Nutrition Year: 2017 Leadership: Led by the government MOHP Approach: Public awareness campaigns on the harmful effects of the consumption of TFA (150)	Name of initiative: GSO 2483/2015 (E) Year: 2016 Leadership: Led by GSO Approach: Mandatory: provides standards which mandate the nutritional labeling of fat, including TFAs, as g/100 g and %DV (50, 111, 112), requiring declaration of TFA as part of nutrition labels for products containing 0.5 g/100 g or more and regulating “trans-fat free” claims (108, 111)	–

*DV, daily value; EMR, Eastern Mediterranean Region; FBDG, food-based dietary guideline; FNA, Food and Nutrition Authority; GCC, Gulf Cooperation Council; GSO, GCC Standardization Organization; JSMO, Jordanian Standards and Metrology Organization; KP, Khyber Pakhtunkhwa; KSA, Kingdom of Saudi Arabia; MOCI, Ministry of Commerce and Industry; MOH, Ministry of Health; MOHAP, Ministry of Health and Prevention; MOHME, Ministry of Health and Medical Education; MOHP, Ministry of Health and Population; MOPH, Ministry of Public Health; NA, not available; NCD, non-communicable disease; NFP, nutrition focal point; NGO, non-governmental organization; PHFS, Patient Helping Fund Society; PHO, partially hydrogenated oil; PSQCA, Pakistan Standard and Quality Control Authority; SFA, saturated fatty acid; SFDA, Saudi Food and Drug Authority; TFA, trans fatty acid; UAE, United Arab Emirates; UFA, unsaturated fatty acid; WHO, World Health Organization*.

### Leadership and Strategic Approach

National strategies or action plans that express a commitment to reduce TFA in the food supply were identified in 12 countries of the region, as shown in Table 2. The identified recommendations and strategies were mainly led by governments, or by national multi-sectoral national committees that include governmental entities, as well as representatives from the food industry, academia and NGOs. In Pakistan, the responsibility for formulating and enacting TFA legislation has recently been transferred from provincial authorities to the Pakistan Standards and Quality Control Authority and a national action plan for industrial TFA elimination is being developed (108, 109). Six countries have specified targets for their population TFA intake (1% of EI or less), including Bahrain, Egypt, Iran, Jordan, KSA, and Morocco. As shown in Table 2, TFA reduction strategies in countries of the region were part of broader strategies or action plans targeting NCD or healthy diets and lifestyle.

**Table 2:** National TFA reduction strategies or action plans identified in the EMR countries.

**Country**	**National strategy and/or action plan**
Bahrain	Reduce intake of TFA to 1% of EI−2015 (Government and food industries) (Action Plan on Reducing the Use of Saturated and Trans Fats in the GCC Countries; Part of Policy for Ensuring the Quality and Sustainability of Health Services 2019–2022)—Information provided by the NFP
Egypt	Replace TFA (<1% of EI) with UFA−2017 (Not adopted yet) (MOHP) (National Multi-sectoral Action Plan for Prevention and Control of Non-communicable Diseases 2017–2021) (152)
Iran	Reduce intake of TFA to a maximum of 1% of EI (MOHME) (National Action Plan for Prevention and Control of Non-communicable Diseases and the Related Risk Factors in the Islamic Republic of Iran 2015–2025, Nutrition and Food Security Policy Statement—MOHME, Part of National Nutrition and Food Security Policy Statement 2015–2025) (41, 63)
Jordan	Reduce intake of TFA to <1% of EI (Government-Multi-sectoral National Committee for Combating Obesity) (Part of National Program for Combating Obesity by Low Fat, Low Sugar, Low Salt)—Information provided by the NFP
	Reduce intake of TFA to <1% of EI−2015 (Government) (National Strategy and Plan of Action Against Diabetes, Hypertension, Dyslipidemia, and Obesity) (127)
KSA	Reduce intake of TFA to <1% of EI−2015 (SFDA) (Healthy Food Strategy) (43, 131, 134)
	Reduce intake of TFA from canned foods to <1% of EI−2015 (MOH) (National Strategy for Healthy Food and Physical Activity 2015–2025) (153)
Kuwait	Lift subsidy on full fat dairy produce, cooking oils and on full fat cheese−2012 (Kuwait's Salt and Fat Intake Reduction Task Force) (Kuwait Action Plan for SFA Intake Reduction and TFA Elimination) (138)
Morocco	Replace TFA in food products with UFA−2015 (MOH) (Non-Communicable Disease Prevention: Multi-sectoral Plan of Action for the Promotion of a Healthy Lifestyle 2015–2020) (154)
	Reduce intake of TFA to 1% of EI−2019 (Planned) (MOH) (Plan to Reduce the Use of Trans Fatty Acids in Processed Products; Part of the Nutrition Program) (49)
Oman	Ban production, importation and marketing of any food containing partially hydrogenated oil (Planned) (Government) (Part of National Nutrition Strategy 2020–2030, National Plan for Prevention of NCD 2016–2025) (155, 156)
Palestine	Avoid the consumption of hydrogenated oils−2019 (MOH) (National Health Strategy 2021–2023) (157)
Qatar	Targets for TFA levels in foods and snacks and taxation on high TFA products−2019 (Planned) (MOPH) (Initiative to Reduce Fat, Sugar and Salt Consumption in Qatar; Part of Qatar Public Health Strategy 2017–2022)—Information provided by the NFP
Tunisia	Replace TFA with UFA and eliminate the intake of TFA−2018 (Not adopted yet) (MOH) (National Multi-sectoral Strategy for the Prevention and Control of Non-Communicable Diseases) (158)
UAE	Replace TFA in food products with UFA−2017 (MOHP) (National Action Plan in Nutrition) (150)

*EI, energy intake; EMR, Eastern Mediterranean Region; GCC, Gulf Cooperation Council; KSA, Kingdom of Saudi Arabia; MOH, Ministry of Health; MOHME, Ministry of Health and Medical Education; MOHP, Ministry of Health and Population; MOPH, Ministry of Public Health; NCD, non-communicable disease; NFP, nutrition focal point; SFA, saturated fatty acids; SFDA, Saudi Food and Drug Authority; TFA, trans fatty acids; UAE, United Arab Emirates; UFA, unsaturated fatty acids*.

### Implementation Strategies

All of the 14 countries listed in Table 1 (100%) are implementing some form of TFA limits or bans, with varying degrees in implementation and policy scope. In addition, except for Egypt, Iraq and Morocco, the other countries are implementing complementary TFA reduction interventions, with the most common being consumer education (10/14 countries; 71%), followed by food labeling (9/14 countries; 64%) and initiatives in specific settings (7/14 countries; 50%). Taxation was the least common implementation strategy. In fact, except for Qatar, where taxation of high TFA products is planned, and for Oman where taxation on the use of hydrogenated oils, was included as part of the National plan for the prevention and control of chronic non-communicable diseases 2016–2025 (not adopted yet) (143), none of the other countries have included taxation-based initiatives. Table 1 displays the details of the initiatives implemented in the various EMR countries.

#### TFA Bans or Limits

Initiatives based on TFA bans or limits included the setting of limits for TFA levels in fats and oils, and other foods (such as bakery products, biscuits, cakes, snacks, and fast foods), and/or the banning or replacement of PHOs.

Iran is the first country in the region to have developed TFA regulation, a process that was initiated in 2005, when the standards for frying oil and mixed liquid oils were revised, setting the maximum level of TFA at 10%, down from over 20% (120). The 2% limit in oils and fats was later adopted, and became effective in 2016 (4, 110). In 2015, the Gulf Cooperation Council (GCC) Standardization Organization (GSO) has set TFA limits of 2% of total fat in vegetable oils and soft spreadable margarines, and 5% of total fat in other foods. The implementation of the regulation took effect in 2016 in Bahrain, while in KSA, UAE and Kuwait, it came into force in 2017. In Qatar, the GSO standard has been submitted to the cabinet in 2020 for endorsement (108), and in Oman, a ministerial decree has been issued stating that the Directorate of Standards should follow all GSO-approved standards.

As for regulating the use of PHO, KSA has become the first country to implement a complete ban on the use of PHO in food products (0 g) in 2020 (108, 110, 128, 129, 132). In Oman a Ministerial Decree to prohibit the use of PHOs is in preparation (155, 156). Iran implements a restriction of PHO use rather than a ban, while Iraq has removed the subsidy on PHOs, and replaced them by other types of oil (14, 50, 113, 122). In Oman, there has been a proposal to tax PHOs, but this has not been adopted or implemented yet (143). Jordan banned the use of PHOs in dairy products (50, 123, 124) and Pakistan has prohibited their use in formula milk (146). Initiatives that included TFA limits/bans were mandatory in 8 countries, including Bahrain, Iran, Jordan, KSA, Kuwait, Morocco, Oman and Palestine (8/14 countries; 57%) (Table 1).

#### Consumer Education

Ten out of the 14 countries (71%) have implemented consumer education campaigns. These countries include Bahrain, Iran, Jordan, KSA, Kuwait, Oman, Palestine, Qatar, Tunisia, and the UAE (Table 1). While most awareness and educational campaigns were led solely by governmental entities, NGOs were collaborators in both Bahrain and Oman. Half of the countries had their consumer education initiatives specific to TFA (Bahrain, Kuwait, Palestine, Tunisia, and the UAE). The other half had broader campaigns in relation to saturated fats, salt, sugar and healthy lifestyle (Iran, Jordan, KSA, Oman, and Qatar).

#### Food Labeling

Nine countries (64%), including Bahrain, Iran, Jordan, KSA, Kuwait, Oman, Pakistan, Qatar and the UAE, were found to have labeling initiatives specific to TFA. These initiatives were mandatory in Bahrain, Iran, KSA, Kuwait, Oman, Qatar and the UAE (7/9 countries; 78%), with the mandatory traffic light labeling scheme being implemented in Iran. In Jordan, and as part of the “National Strategy and Plan of Action Against Diabetes, Hypertension, Dyslipidemia and Obesity,” labeling of food products with TFA was set to be mandatory as of 2015, however this initiative has not been adopted yet (4, 127) (Table 1).

#### Interventions in Specific Settings

Seven countries (50%) are implementing TFA reduction initiatives in specific settings. These include Bahrain, Iran, Jordan, KSA, Kuwait, Oman and Qatar. Bahrain is targeting school canteens through the use of a prohibited food list and by introducing modifications to food preparation methods. Iran is implementing TFA educational interventions in schools and workplaces, while in Jordan, TFA reduction interventions are being implemented in public hospitals and royal medical services (108). Under the Nutrition-Friendly Schools Initiative, Kuwaiti schools are being targeted to raise nutrition and health awareness, with a focus on TFA intake reduction (136, 140). In Qatar, the adoption of the School Canteen Guidelines is mandatory in governmental schools, while the implementation of educational sessions in workplaces remain voluntary.

Other initiatives that have been implemented and shared with various governmental entities in KSA (hospitals, universities, military, or public places) include the replacement of PHOs and saturated fatty acids with unsaturated fatty acids (UFA) in offered foods (135). Similarly, the elimination of TFA in foods offered in governmental institutions in Kuwait has been proposed, although not adopted yet (138). In Oman, and as part of the National Plan for the Prevention and Control of Chronic Non-communicable Diseases 2016–2025, limiting high TFA items in schools is planned, but has not been implemented yet (143) (Table 1). Interventions or policy actions targeting the food service sector (such as restaurants and coffee-shops) were not identified in any country of the region.

### Monitoring and Evaluation

Monitoring activities are being conducted in Iran and several GCC countries including Bahrain, KSA, Kuwait, and Qatar. In Iran, post marketing surveillance is conducted by the Food and Drug Organization on an annual basis. Accordingly, samples of edible oils and fats (frying oil, consumer edible vegetable oil, Margarine, minarine, and shortening), are analyzed to determine TFA levels (40). The Iran Standard Organization also monitors the TFA content in bakery products, biscuits and confectionary products. Ghazavi et al. collected samples of traditional sweets with nutritional labeling, and compared the TFA information on the label with actual TFA levels based on chemical analysis. Results showed that there was 81.8% discrepancy between the analytical levels and those listed on the label (65).

In Bahrain, monitoring activities focus on the monitoring of TFA content in samples of bakery products, and fats and oils via direct chemical analyses. Moreover, the listing and proper labeling of TFA on prepackaged products (both locally produced and imported) is being monitored in Bahrain. In KSA, the SFDA, in collaboration with the Ministry of Municipal and Rural Affairs, is responsible for monitoring and inspection activities, targeting food products that are locally produced or imported. Laboratory analyses are conducted in the SFDA laboratories. In 2020, SFDA launched a campaign to ensure that manufacturers and importers of food products comply with the Saudi Technical Regulation SFDA.FD 2483 “Trans fats (fatty acids).” The inspection focused on local and imported food products such as margarine, donuts, cakes, biscuits, pies, frozen pastries, cheese, chocolates, ice cream, and other items. The total number of samples that were chemically analyzed was of 2,697 (129, 159), and the percentage of violation was estimated at 20% (200 local products and 332 imported products). An older campaign conducted in 2018 to monitor compliance to TFAs limit in food products, showed that 94% of the 400 samples that were tested were compliant with the standards (110). In 2016–2017, Jradi et al. (76) conducted a study to assess compliance with SFDA nutritional facts requirements: only 38% met the SFDA requirements for nutritional information (energy, protein, carbohydrate, sugar, total fat, saturated fat, TFA, and sodium). Among the missing nutrients, TFA was the most frequently omitted (54.5%) from the nutritional facts.

In Kuwait, the National Technical Food Committee has developed a plan to monitor the implementation of the TFA standards (136). In collaboration with the food laboratory—Ministry of Health (MOH)- measures have been put in place to monitor, inspect and evaluate the compliance of the food industry by chemically analyzing TFA content in randomly selected samples of potentially high TFA food sources. In this context, a capacity-building program has been conducted by the Department of Standards and Inspection to enhance the inspectors' capabilities, and assure compliance of the food industry with the various GSO standards and technical regulations (Trans Fatty Acids GSO 2483/2015; Requirements of nutritional labeling. GSO 2233/2012; Nutritional and Health Claims Requirements. GSO 2333/2013). In addition, the TFA content declared on the nutrition facts is monitored to evaluate compliance with the technical regulation for TFA.

In Qatar, the Health Promotion and NCD Section of the Public Health Department at the Ministry of Public Health plays a role in the development of the School Canteen Guidelines, led by the Ministry of Education and Higher Education. The Health Promotion and NCD section of the Public Health Department at the MOPH has also developed and implemented the Food & Beverage Guidelines in Cafeterias and Vending Machines in all healthcare settings and workplaces that are part of the Workplace Wellness Program. An annual evaluation tool is administered to all hospitals and workplaces that are implementing the guidelines.

### Impact Assessment

Very few countries have assessed the impact of TFA reduction initiatives on the population's intake levels. In addition, the majority of countries have only one estimate of TFA intake, and therefore the investigation of trend in TFA intake is not possible. The two exceptions are Iran and UAE. In Iran, the intake of TFA for the entire population (per capita) was estimated at 4.2% EI in 2001–2003, i.e., prior to policy implementation (39). This intake had decreased to 0.7% in 2018 as reported by Amerzadeh and Takian (41), post policy implementation. In the UAE, a study conducted amongst female university students had estimated TFA intake at 1.7% in 2014 (14) before the implementation of TFA reduction initiatives. Intake level has decreased to 1% in 2017–2018 in the same population group, post policy implementation (51).

Data on the impact of TFA reduction initiatives on the levels of TFA in food products is also scarce. Available evidence suggests that the legislations that were implemented in Iran resulted in a significant reduction in the level of TFAs content in edible oil. In fact, initiatives aimed at reducing the level of TFA in edible oils were launched in 2005, when the standards for frying oil and mixed liquid oils were revised to reduce the maximum level of TFA (to <10%, down from over 20%) (120), and when the Ministry of Commerce started the gradual replacement of hydrogenated oils by non-hydrogenated (specifically olive oil) and liquid frying oils (14, 50, 113). Peymani et al. assessed the impact of this policy on the levels of TFA in edible oils. Samples were collected randomly over a 6-year period, from 2002 to 2008, from different national edible oil manufacturers. Tests were conducted by the national referral laboratory in <city>Tehran</city>, Iran. Results showed that the TFA composition (%) of edible oil represented 27–29% in 2002–2003, 31.2% in 2004–2005, decreasing to 13.7% in 2007 and 5.6% in 2008 (121). Another study was conducted in 2014 by the Post Marketing Surveillance (PMS) in Iran: It targeted households in six provinces of Iran, whereby information on the types of fats and oils used for cooking as well as the purchase pattern and their amounts were obtained using questionnaires. In addition, the fatty acid profiles of consumed fats and oils were determined (40). The analysis of TFA in different kinds of oils (frying oil, consumer edible vegetable oil, margarine, minarine, and shortening) was performed. Results showed that the TFA content of edible oils has been reduced to <5% TFAs (40).

## Discussion

This systematic review is the first to focus on TFA reduction initiatives in the EMR, a region that is currently witnessing an increasing burden of cardiovascular morbidity and mortality (13). It showed that out of the 22 countries of the EMR, nine have assessed TFA intake in the population (41%), nine (41%) have evaluated TFA levels in food items, and fourteen (63.6%) have implemented national TFA reduction initiatives. The most common TFA reduction initiatives were based on TFA limits or bans in an effort to decrease/eliminate TFAs from the food supply, while the least common was taxation.

Based on available national studies conducted in countries of the region, the population's TFA intake levels were found to range between 0.34% EI in Morocco and 6.5% EI in Egypt (4, 49), while subnational studies reported estimates ranging between 0.1% EI in Sudan and 2.3–2.4% EI in Lebanon (14, 48). Available data highlight very high intake levels in Egypt (6.5% EI) (4) and Pakistan (5.7% EI) (4). These estimates are 5–6 fold higher than the upper limit of 1% EI specified by the WHO (160, 161) and the American Heart Association (162), and significantly higher that the optimal level defined by the Global Burden of Disease (GBD) collaborators, in their evaluation of dietary risk factors (0.5% EI, with an optimal range of 0–1% EI) (163). Based on the 2019 GBD data, the Institute for Health Metrics and Evaluation (IHME) has assessed the burden of CHD attributable to high TFA intake (>0.5% EI) in each country worldwide (4, 12). Accordingly, the top ten countries included three from the EMR: Egypt was ranked at the top of the list as the country with the highest burden of CHD due to high TFA intake in the world (8.4% of CHD deaths), Iran ranked as the third (6.96% of CHD deaths) and Pakistan as the ninth (4.94% of CHD deaths) (12).

Countries that had very low estimates of TFA intake included Morocco (0.34% EI) (49), Jordan (0.5–0.6% EI) (14) and Sudan (0.1% EI) (14). It is important to mention that dietary estimates of TFA intake should be interpreted with caution. These are in fact limited by the available food composition databases that either have a large number of missing data for TFA levels in foods, or are not culture-specific (164). Without country- or region-specific data, TFA elimination would often not be recognized as a priority for time and resource investment (12). Acknowledging this challenge, the WHO has published a standardized protocol for TFA analysis (98) that can be adapted for measuring TFA levels in national food supplies in different settings (12), and the WHO EMRO has supported the launch of TFA analysis projects in several Member States. This endeavor has been already completed in Egypt, Lebanon, Morocco (data not published yet), and Jordan (94). Such data should help in enhancing the robustness and accuracy of future TFA dietary assessment in countries of the region.

Very few studies have reported on TFA intake amongst children and adolescents in the EMR, although evidence suggests that this age group may have the highest TFA intake level given their tendency to consume “fashionable” processed foods as well as fast foods (165). In Iran, TFA intake amongst 6–18 year old children and adolescents was reported at 2.2–2.3% EI in 2019 (43). This estimate highlights that TFA intake remains high in this age group, even after the implementation of TFA reduction initiatives which have been launched as of 2005 (4, 14, 41, 50, 57, 105, 108, 110, 113–121). More attention should be devoted to this age group, and their food consumption patterns, since high TFA intakes in this period of the lifecycle may increase the risk for early onset CVD and related comorbidities (165).

The number of countries adopting TFA limits or bans to reduce TFA levels in the food supply is estimated at 14 (63.6%), with significant disparities between countries in policy scope and coverage. The main disparities were noted in whether the limits/bans were mandatory or not (8 mandatory vs. 6 voluntary), and the number and types of foods targeted by the standards. Iran is the first country to have adopted TFA limits and regulations in the region, while KSA is the first to have implemented a best-practice policy according to the WHO (12). A best-practice policy is defined as “Legislative or regulatory measures that limit industrially produced TFA in foods in all setting. The two best-practice policies for TFA elimination are: Mandatory national limit of 2 g of industrially produced TFA per 100 g of total fat in all foods; and Mandatory national ban on the production or use of PHO as an ingredient in all foods.” Countries of the GCC have all adopted or are on the way of implementing the GSO standards for TFA which set TFA limits at 2% of total fat in vegetable oils and soft spreadable margarines, and 5% of total fat in other foods (12). More work needs to be done in the other EMR countries that do not have any TFA replacement strategy. In fact, policy interventions to eliminate industrial TFAs from food have been proposed as the most effective public health approach for reducing TFA intake and reducing the burden of NCDs (166). Based on several modeling studies, a recent systematic review showed that policies that set TFA limits are likely to be cost-saving in addition to having the greatest impact on lower socioeconomic groups (9). These are vital considerations for policymakers in countries of the region in terms of deciding whether or not to adopt a TFA reduction policy (9). Downs et al. argued that TFA bans make sense from both an economic standpoint as well as an ethical perspective, given that they could contribute to reducing social inequities (9). It is however also acknowledged that the adoption of a TFA removal policy will also require substantial political commitment and a high level of public pressure for change, which may not be readily available or achievable in many countries of the region (6).

Besides TFA limits or bans, complementary TFA reduction approaches have been implemented in several countries of the region. This is a positive finding given that multicomponent interventions, including a legislative ban on high TFA products, may increase the impact and effectiveness of TFA reduction strategies (6). Consumer education was the most common complementary approach in countries of the EMR, with the aim of raising awareness about TFA, its main dietary sources and its adverse health effects. For instance, the reduction of TFA intake has been included in country-specific food-based dietary guidelines in Jordan and Qatar. Such guidelines and their communication to the public has been recognized as a practical and effective approach in improving the consumer's dietary knowledge and attitudes (167, 168). While none of the countries had assessed consumers' attitude toward TFA, the few available studies that have assessed consumers' knowledge had in fact shown that consumers have poor or little knowledge related to the sources of TFA (101) and the potential health effects of excessive TFA consumption (102). From the few available studies on practices, one study conducted in KSA, has shown that the vast majority of adolescents and adults are not interested in checking TFA-related information on the food label (75). While, few studies have shown that a large percentage of consumers (31.5–45%) continue to use high TFA sources (e.g., hydrogenated vegetable oils) in Iran and Pakistan (99, 105, 106), other studies reported much smaller estimates (0–7.3%) in Iran, Iraq, KSA and the UAE (100, 103, 104, 107). It is recommended that countries of the region consult findings stemming from the available knowledge and practice investigations when developing or further tailoring their consumer education initiatives in order to address culture-specific gaps in knowledge and identify barriers against TFA reduction. Acknowledging that labeling can be an important element of national strategies aimed at improving the population's diets and reducing TFA intake (6), a promising finding of this review is the fact that TFA-specific labeling initiatives are being implemented or planned in 9 countries of the region. Of these, front of pack labeling, which is recognized as being easily understood by the consumer (169), is being implemented in Iran. The fact that taxation was the least implemented/adopted in the region is in line with reports from other parts of the world (6).

This review showed that a number of countries have included a legislative component within their TFA reduction strategies, instead of implementing solely voluntary initiatives. Previous studies have shown that mandatory or legislative approaches tend to be more effective, producing more significant reductions in TFA intake levels within the population, compared to voluntary approaches (6). A recent systematic review has concluded that, although all policy approaches may lead to reductions in TFA levels in foods and subsequent intakes, stronger policies (i.e., mandatory TFA limits or bans) will have more pronounced effects than voluntary food reformulation or labeling approaches (6).

The implementation of clear monitoring activities is essential to demonstrate program effectiveness, and to incite greater impact on TFA reduction (12). In the EMR, five countries only (Iran, Bahrain, KSA, Kuwait, and Qatar) have established mechanisms for the monitoring of TFA content in edible oils, fats, bakery and snack products, using laboratory analyses. The lack of laboratory capacity to perform TFA measurements in foods, especially in lower-resourced countries, may in fact be a barrier to monitoring and enforcement (12). Some countries in the region have mandatory labeling (TFA declaration on nutrient facts panels and/ or PHO on the ingredients list), which should ensure compliance with regulations. However, even when labeling requirements are already in place, it is crucial for countries to be able to confirm, through laboratory testing, that the food industry is complying with the information on the labels. For those countries that do not have mandatory labeling requirements, these have to rely solely on laboratory testing of TFA levels to monitor compliance. More work needs to be done to support countries, particularly in low resource settings, in conducting TFA laboratory analyses. An additional measure that can be explored is the identification of laboratories in the region that have demonstrated capacities for analyzing TFAs in foods and which can support laboratory testing in neighboring countries (12).

Data on the impact of TFA reduction initiatives is scarce in the region. Except for Iran and the UAE, where there is evidence of a decrease in the population's TFA intake after the implementation of TFA reduction strategies (14, 39, 41, 51), none of the other countries have conducted impact evaluation. The impact on TFA levels in foods is also sparsely described, with only Iran having provided such evidence (41, 116, 170). The scarcity of data may be partially due to the fact that many reduction initiatives in the region are relatively recent and there has been insufficient time to assess impact. There is a need for well-designed impact evaluations in countries of the region. The WHO analytical protocol for TFA analysis (98) provides a valuable tool for such undertakings, allowing for the implementation of standardized methodologies in the analysis of TFA in foods and hence the documentation of change in TFA levels over time. Standardized comparable dietary approaches to measuring TFA intake in the population are also needed. Although dietary approaches carry their own inherent limitations in TFA intake assessment (164), if the adopted method of assessment is consistent, it can still be a useful measure of change over time (171, 172). Since the regular measurement of changes in population TFA intake and in TFA levels in foods may be complex and costly, the incorporation of process evaluations that examine the strategy implementation and its progress, collects process indicators, and identifies existing barriers and facilitators of implementation is also vital in providing real-time information and identifying specific areas for improvement (173).

This review has a number of strengths and limitations. This is the first systematic review of existing TFA reduction initiatives in countries of the EMR, their implementation and progress over time. In addition to the systematic search of databases and gray literature, additional input was sought from focal points or program leaders in the various countries that were identified as having existing TFA reduction initiatives, to verify and obtain supplementary country-specific data. Although not all country contacts were identified and there were some non-respondents, the triangulation of data from multiple sources allowed us to document the existing initiatives and implementation of strategies, and present the information in a relatively standardized manner. Through this, it is unlikely that any major TFA reduction initiatives were omitted, although this possibility cannot be totally disregarded. While one of the major strengths of the review is the fact that it included a comprehensive search of the gray literature, comprising governmental reports, presentations or questionnaires completed by country program leaders, a potential limitation associated with this approach is the fact that the methodological rigor within some of the reports is not ascertained. More specifically, the robustness of the studies, their methodology and the quality of the data used for the assessment of TFA intake and TFA levels in foods were not assessed and hence should be interpreted with caution. It is also important to note that studies that have reported on TFA intake were scarce and that dietary estimation of TFA intake levels is by itself limited by the scarcity of up-to date, culture-specific food composition databases.

## Conclusion

Despite the ongoing TFA reduction initiatives in several countries of the region, this study showed that intake estimates from several countries of the EMR remain high, exceeding the WHO upper limit of 1%. This review has also shown that 14 countries (63.6%) have adopted TFA limits or bans to reduce TFA levels in the food supply, albeit with significant disparities between countries in policy scope and coverage. It is recommended that all countries of the region align with the global best practice policies by implementing a ban on PHO and setting mandatory national limit of 2 g of industrially produced TFA per 100 g of total fat in all foods (12). Further action is needed to make sure that countries strengthen their regulatory capacities to help accelerate implementation, compliance monitoring and enforcement of TFA policy actions, and meet the targeted elimination of industrially produced TFA in 2023.

## Data Availability Statement

The original contributions presented in the study are included in the article/Supplementary Material, further inquiries can be directed to the corresponding author/s.

## Author Contributions

AA-J and LN: conceptualization, investigation, resources, and supervision. LN: methodology and writing—original draft preparation. SN: systematic database search. MT, AC, and LN: data curation. MT and AA-J: writing—review and editing. AA-J: project administration. ZA, BA, NA, MMA, SA, RA, SAA, MHA, NB, RB, FB, LE, JE, MH, HM, and MN: critical review of country-specific data and of manuscript. All authors have read and agreed to the published version of the manuscript.

## Conflict of Interest

The authors declare that the research was conducted in the absence of any commercial or financial relationships that could be construed as a potential conflict of interest.

## Publisher's Note

All claims expressed in this article are solely those of the authors and do not necessarily represent those of their affiliated organizations, or those of the publisher, the editors and the reviewers. Any product that may be evaluated in this article, or claim that may be made by its manufacturer, is not guaranteed or endorsed by the publisher.

## Abbreviations

CHD: coronary heart disease
CVD: cardiovascular diseases
DV: daily value
EI: energy intake
EMR: Eastern Mediterranean Region
EMRO: Regional Office for the Eastern Mediterranean
FBDG: food-based dietary guideline
FNA: Food and nutrition authority
GBD: global burden of disease
GCC: Gulf Cooperation Council
GINA: Global database on the implementation of nutrition action
GSO: GCC standardization organization
HDL: high-density lipoprotein
KAB, knowledge: attitudes and behavior
KP: Khyber Pakhtunkhwa
KSA: Kingdom of Saudi Arabia
LDL: low-density lipoprotein
MOCI: Ministry of commerce and industry
MOH: Ministry of health
MOHAP: Ministry of health and prevention
MOHME: Ministry of health and medical education
MOHP: Ministry of health and population
MOPH: Ministry of public health
NA: not available
NCD: non-communicable disease
NFP: nutrition focal point
NGO: non-governmental organization
PHFS: Patient helping fund society
PHOs: partially hydrogenated oils
PMS: post marketing surveillance
PSQCA: Pakistan standard and quality control authority
SFA: Saturated fatty acid
SFDA: Saudi food and drug authority
TFA: Trans fatty acid
UAE: United Arab Emirates
UFA: Unsaturated fatty acid
WHO: World Health Organization.

## Appendix

**Table A1:** List of search terms.

Trans fats	“trans fat*” OR TFA OR trans fatty acids OR “partially hydrogenated oil*” OR “partially hydrogenated fat*” OR “partially hydrogenated vegetable oil*”
AND	
Reduction	reduce* OR reduction* OR reducing OR decreas* OR limit OR limits OR limitation* OR limiting OR restrict* OR reformulat* OR low*
OR	
Intake	consumption OR consuming OR consume OR consumes OR intake* OR food* OR nutrition OR diet*
AND	
Strategy/policy	standard* OR polic* OR initiative* OR tax* OR program* OR regulation* OR strateg* OR guideline* OR practice* OR legislat* OR action* OR plan OR plans OR intervention* OR law* OR campaign* OR marketing OR advertise* OR label* OR incentive* OR ban* OR recommendation*
AND	
EMR	Afghan* OR Bahrain* OR Iran* OR Persia* OR Iraq* OR Jordan* OR Kuwait* OR Lebanon* OR Lebanese OR Libya* OR Oman* OR Palestin* OR Gaza* OR “West Bank” OR Qatar* OR Saud* OR KSA OR Syria* OR Tunis* OR “United Arab Emirate*” OR UAE OR Djibouti* OR Egypt* OR Morocc* OR Pakistan* OR Somal* OR Sudan* OR Yemen* OR Levant* OR “East* Mediterranean” OR Gulf OR GCC OR Arab OR Arabia OR Arabs OR EMR OR “Middle East*” OR MENA OR “North* Africa*” OR “East* Africa*” OR “Near East*” OR “Abu Dhabi” OR Dubai OR Ajman OR Fujaira* OR Sharja* OR *Khaima* OR *Qaiwain OR *Quwain

*EMR, Eastern Mediterranean Region; GCC, Gulf Cooperation Council; KSA, Kingdom of Saudi Arabia; MENA, Middle East and North Africa; TFA, trans fatty acid; UAE, United Arab Emirates*.
