# Supplementary material for: A Systematic Review of Trans Fat Reduction Initiatives in the Eastern Mediterranean Region
Source: Front Nutr. 2021 Nov 26;8:771492. doi: 10.3389/fnut.2021.771492 (PMC8662545; doi:10.3389/fnut.2021.771492)
Supplement: Supplementary file 1 [file Table_1.DOCX]

**Supplementary Table 1.** **Example of a Database Search.**

| **Search Number** | **Query** | **Filters** | **Results** |
| --- | --- | --- | --- |
| 13 | ((("Africa, Eastern"[Mesh:NoExp] OR "Djibouti"[Mesh] OR "Somalia"[Mesh] OR "South Sudan"[Mesh] OR "Sudan"[Mesh] OR "Africa, Northern"[Mesh:NoExp] OR "Egypt"[Mesh] OR "Libya"[Mesh] OR "Morocco"[Mesh] OR "Tunisia"[Mesh] OR "Middle East"[Mesh:NoExp] OR "Afghanistan"[Mesh] OR "Bahrain"[Mesh] OR "Iran"[Mesh] OR "Iraq"[Mesh] OR "Jordan"[Mesh] OR "Kuwait"[Mesh] OR "Lebanon"[Mesh] OR "Oman"[Mesh] OR "Qatar"[Mesh] OR "Saudi Arabia"[Mesh] OR "Syria"[Mesh] OR "United Arab Emirates"[Mesh] OR "Yemen"[Mesh] OR "Pakistan"[Mesh]) OR (Afghan*[tiab] OR Bahrain*[tiab] OR Egypt*[tiab] OR Iran*[tiab] OR Persia*[tiab] OR Iraq*[tiab] OR Jordan*[tiab] OR Kuwait*[tiab] OR Lebanese[tiab] OR Lebanon[tiab] OR Oman*[tiab] OR Palestin*[tiab] OR Gaza*[tiab] OR "West Bank"[tiab] OR Qatar*[tiab] OR Saudi*[tiab] OR KSA[tiab] OR Syria*[tiab] OR "Tunis*"[tiab] OR "United Arab Emirates"[tiab] OR UAE[tiab] OR "Abu Dhabi"[tiab] OR Dubai[tiab] OR Ajman[tiab] OR Fujaira*[tiab] OR Sharja*[tiab] OR Khaima*[tiab] OR Qaiwain[tiab] OR Quwain[tiab] OR Yemen*[tiab] OR Libya*[tiab] OR Djibouti*[tiab] OR Morocc*[tiab] OR Pakistan*[tiab] OR Somal*[tiab] OR Sudan*[tiab] OR "Middle East"[tiab] OR "Middle Eastern"[tiab] OR Arab[tiab] OR Arabic[tiab] OR Arabs[tiab] OR Arabia[tiab] OR "Near East"[tiab] OR "Near Eastern"[tiab] OR Levant*[tiab] OR MENA[tiab] OR EMR[tiab] OR "East Mediterranean"[tiab] OR "Eastern Mediterranean"[tiab] OR Gulf[tiab] OR GCC[tiab] OR "North Africa"[tiab] OR "North African"[tiab] OR "Northern Africa"[tiab] OR "Northern African"[tiab] OR "East Africa"[tiab] OR "East African"[tiab] OR "Eastern Africa"[tiab] OR "Eastern African"[tiab])) AND (consumption[tiab] OR consuming[tiab] OR consume[tiab] OR consumes[tiab] OR intake*[tiab] OR food*[tiab] OR nutrition[tiab] OR diet*[tiab] OR source*[tiab])) AND ((((("Trans Fatty Acids"[Mesh]) OR (transfat*[tiab])) OR (trans-fat*[tiab])) OR (TFA[tiab])) OR ("partially hydrogenated"[tiab])) | Arabic, English, French, from 1995 - 2021 | 64 |
| 12 | ((("Africa, Eastern"[Mesh:NoExp] OR "Djibouti"[Mesh] OR "Somalia"[Mesh] OR "South Sudan"[Mesh] OR "Sudan"[Mesh] OR "Africa, Northern"[Mesh:NoExp] OR "Egypt"[Mesh] OR "Libya"[Mesh] OR "Morocco"[Mesh] OR "Tunisia"[Mesh] OR "Middle East"[Mesh:NoExp] OR "Afghanistan"[Mesh] OR "Bahrain"[Mesh] OR "Iran"[Mesh] OR "Iraq"[Mesh] OR "Jordan"[Mesh] OR "Kuwait"[Mesh] OR "Lebanon"[Mesh] OR "Oman"[Mesh] OR "Qatar"[Mesh] OR "Saudi Arabia"[Mesh] OR "Syria"[Mesh] OR "United Arab Emirates"[Mesh] OR "Yemen"[Mesh] OR "Pakistan"[Mesh]) OR (Afghan*[tiab] OR Bahrain*[tiab] OR Egypt*[tiab] OR Iran*[tiab] OR Persia*[tiab] OR Iraq*[tiab] OR Jordan*[tiab] OR Kuwait*[tiab] OR Lebanese[tiab] OR Lebanon[tiab] OR Oman*[tiab] OR Palestin*[tiab] OR Gaza*[tiab] OR "West Bank"[tiab] OR Qatar*[tiab] OR Saudi*[tiab] OR KSA[tiab] OR Syria*[tiab] OR "Tunis*"[tiab] OR "United Arab Emirates"[tiab] OR UAE[tiab] OR "Abu Dhabi"[tiab] OR Dubai[tiab] OR Ajman[tiab] OR Fujaira*[tiab] OR Sharja*[tiab] OR Khaima*[tiab] OR Qaiwain[tiab] OR Quwain[tiab] OR Yemen*[tiab] OR Libya*[tiab] OR Djibouti*[tiab] OR Morocc*[tiab] OR Pakistan*[tiab] OR Somal*[tiab] OR Sudan*[tiab] OR "Middle East"[tiab] OR "Middle Eastern"[tiab] OR Arab[tiab] OR Arabic[tiab] OR Arabs[tiab] OR Arabia[tiab] OR "Near East"[tiab] OR "Near Eastern"[tiab] OR Levant*[tiab] OR MENA[tiab] OR EMR[tiab] OR "East Mediterranean"[tiab] OR "Eastern Mediterranean"[tiab] OR Gulf[tiab] OR GCC[tiab] OR "North Africa"[tiab] OR "North African"[tiab] OR "Northern Africa"[tiab] OR "Northern African"[tiab] OR "East Africa"[tiab] OR "East African"[tiab] OR "Eastern Africa"[tiab] OR "Eastern African"[tiab])) AND (consumption[tiab] OR consuming[tiab] OR consume[tiab] OR consumes[tiab] OR intake*[tiab] OR food*[tiab] OR nutrition[tiab] OR diet*[tiab] OR source*[tiab])) AND ((((("Trans Fatty Acids"[Mesh]) OR (transfat*[tiab])) OR (trans-fat*[tiab])) OR (TFA[tiab])) OR ("partially hydrogenated"[tiab])) | from 1995 - 2021 | 64 |
| 11 | ((("Africa, Eastern"[Mesh:NoExp] OR "Djibouti"[Mesh] OR "Somalia"[Mesh] OR "South Sudan"[Mesh] OR "Sudan"[Mesh] OR "Africa, Northern"[Mesh:NoExp] OR "Egypt"[Mesh] OR "Libya"[Mesh] OR "Morocco"[Mesh] OR "Tunisia"[Mesh] OR "Middle East"[Mesh:NoExp] OR "Afghanistan"[Mesh] OR "Bahrain"[Mesh] OR "Iran"[Mesh] OR "Iraq"[Mesh] OR "Jordan"[Mesh] OR "Kuwait"[Mesh] OR "Lebanon"[Mesh] OR "Oman"[Mesh] OR "Qatar"[Mesh] OR "Saudi Arabia"[Mesh] OR "Syria"[Mesh] OR "United Arab Emirates"[Mesh] OR "Yemen"[Mesh] OR "Pakistan"[Mesh]) OR (Afghan*[tiab] OR Bahrain*[tiab] OR Egypt*[tiab] OR Iran*[tiab] OR Persia*[tiab] OR Iraq*[tiab] OR Jordan*[tiab] OR Kuwait*[tiab] OR Lebanese[tiab] OR Lebanon[tiab] OR Oman*[tiab] OR Palestin*[tiab] OR Gaza*[tiab] OR "West Bank"[tiab] OR Qatar*[tiab] OR Saudi*[tiab] OR KSA[tiab] OR Syria*[tiab] OR "Tunis*"[tiab] OR "United Arab Emirates"[tiab] OR UAE[tiab] OR "Abu Dhabi"[tiab] OR Dubai[tiab] OR Ajman[tiab] OR Fujaira*[tiab] OR Sharja*[tiab] OR Khaima*[tiab] OR Qaiwain[tiab] OR Quwain[tiab] OR Yemen*[tiab] OR Libya*[tiab] OR Djibouti*[tiab] OR Morocc*[tiab] OR Pakistan*[tiab] OR Somal*[tiab] OR Sudan*[tiab] OR "Middle East"[tiab] OR "Middle Eastern"[tiab] OR Arab[tiab] OR Arabic[tiab] OR Arabs[tiab] OR Arabia[tiab] OR "Near East"[tiab] OR "Near Eastern"[tiab] OR Levant*[tiab] OR MENA[tiab] OR EMR[tiab] OR "East Mediterranean"[tiab] OR "Eastern Mediterranean"[tiab] OR Gulf[tiab] OR GCC[tiab] OR "North Africa"[tiab] OR "North African"[tiab] OR "Northern Africa"[tiab] OR "Northern African"[tiab] OR "East Africa"[tiab] OR "East African"[tiab] OR "Eastern Africa"[tiab] OR "Eastern African"[tiab])) AND (consumption[tiab] OR consuming[tiab] OR consume[tiab] OR consumes[tiab] OR intake*[tiab] OR food*[tiab] OR nutrition[tiab] OR diet*[tiab] OR source*[tiab])) AND ((((("Trans Fatty Acids"[Mesh]) OR (transfat*[tiab])) OR (trans-fat*[tiab])) OR (TFA[tiab])) OR ("partially hydrogenated"[tiab])) | -- | 67 |
| 10 | ("Africa, Eastern"[Mesh:NoExp] OR "Djibouti"[Mesh] OR "Somalia"[Mesh] OR "South Sudan"[Mesh] OR "Sudan"[Mesh] OR "Africa, Northern"[Mesh:NoExp] OR "Egypt"[Mesh] OR "Libya"[Mesh] OR "Morocco"[Mesh] OR "Tunisia"[Mesh] OR "Middle East"[Mesh:NoExp] OR "Afghanistan"[Mesh] OR "Bahrain"[Mesh] OR "Iran"[Mesh] OR "Iraq"[Mesh] OR "Jordan"[Mesh] OR "Kuwait"[Mesh] OR "Lebanon"[Mesh] OR "Oman"[Mesh] OR "Qatar"[Mesh] OR "Saudi Arabia"[Mesh] OR "Syria"[Mesh] OR "United Arab Emirates"[Mesh] OR "Yemen"[Mesh] OR "Pakistan"[Mesh]) OR (Afghan*[tiab] OR Bahrain*[tiab] OR Egypt*[tiab] OR Iran*[tiab] OR Persia*[tiab] OR Iraq*[tiab] OR Jordan*[tiab] OR Kuwait*[tiab] OR Lebanese[tiab] OR Lebanon[tiab] OR Oman*[tiab] OR Palestin*[tiab] OR Gaza*[tiab] OR "West Bank"[tiab] OR Qatar*[tiab] OR Saudi*[tiab] OR KSA[tiab] OR Syria*[tiab] OR "Tunis*"[tiab] OR "United Arab Emirates"[tiab] OR UAE[tiab] OR "Abu Dhabi"[tiab] OR Dubai[tiab] OR Ajman[tiab] OR Fujaira*[tiab] OR Sharja*[tiab] OR Khaima*[tiab] OR Qaiwain[tiab] OR Quwain[tiab] OR Yemen*[tiab] OR Libya*[tiab] OR Djibouti*[tiab] OR Morocc*[tiab] OR Pakistan*[tiab] OR Somal*[tiab] OR Sudan*[tiab] OR "Middle East"[tiab] OR "Middle Eastern"[tiab] OR Arab[tiab] OR Arabic[tiab] OR Arabs[tiab] OR Arabia[tiab] OR "Near East"[tiab] OR "Near Eastern"[tiab] OR Levant*[tiab] OR MENA[tiab] OR EMR[tiab] OR "East Mediterranean"[tiab] OR "Eastern Mediterranean"[tiab] OR Gulf[tiab] OR GCC[tiab] OR "North Africa"[tiab] OR "North African"[tiab] OR "Northern Africa"[tiab] OR "Northern African"[tiab] OR "East Africa"[tiab] OR "East African"[tiab] OR "Eastern Africa"[tiab] OR "Eastern African"[tiab]) | -- | 296,091 |
| 9 | Afghan*[tiab] OR Bahrain*[tiab] OR Egypt*[tiab] OR Iran*[tiab] OR Persia*[tiab] OR Iraq*[tiab] OR Jordan*[tiab] OR Kuwait*[tiab] OR Lebanese[tiab] OR Lebanon[tiab] OR Oman*[tiab] OR Palestin*[tiab] OR Gaza*[tiab] OR "West Bank"[tiab] OR Qatar*[tiab] OR Saudi*[tiab] OR KSA[tiab] OR Syria*[tiab] OR "Tunis*"[tiab] OR "United Arab Emirates"[tiab] OR UAE[tiab] OR "Abu Dhabi"[tiab] OR Dubai[tiab] OR Ajman[tiab] OR Fujaira*[tiab] OR Sharja*[tiab] OR Khaima*[tiab] OR Qaiwain[tiab] OR Quwain[tiab] OR Yemen*[tiab] OR Libya*[tiab] OR Djibouti*[tiab] OR Morocc*[tiab] OR Pakistan*[tiab] OR Somal*[tiab] OR Sudan*[tiab] OR "Middle East"[tiab] OR "Middle Eastern"[tiab] OR Arab[tiab] OR Arabic[tiab] OR Arabs[tiab] OR Arabia[tiab] OR "Near East"[tiab] OR "Near Eastern"[tiab] OR Levant*[tiab] OR MENA[tiab] OR EMR[tiab] OR "East Mediterranean"[tiab] OR "Eastern Mediterranean"[tiab] OR Gulf[tiab] OR GCC[tiab] OR "North Africa"[tiab] OR "North African"[tiab] OR "Northern Africa"[tiab] OR "Northern African"[tiab] OR "East Africa"[tiab] OR "East African"[tiab] OR "Eastern Africa"[tiab] OR "Eastern African"[tiab] | -- | 269,050 |
| 8 | "Africa, Eastern"[Mesh:NoExp] OR "Djibouti"[Mesh] OR "Somalia"[Mesh] OR "South Sudan"[Mesh] OR "Sudan"[Mesh] OR "Africa, Northern"[Mesh:NoExp] OR "Egypt"[Mesh] OR "Libya"[Mesh] OR "Morocco"[Mesh] OR "Tunisia"[Mesh] OR "Middle East"[Mesh:NoExp] OR "Afghanistan"[Mesh] OR "Bahrain"[Mesh] OR "Iran"[Mesh] OR "Iraq"[Mesh] OR "Jordan"[Mesh] OR "Kuwait"[Mesh] OR "Lebanon"[Mesh] OR "Oman"[Mesh] OR "Qatar"[Mesh] OR "Saudi Arabia"[Mesh] OR "Syria"[Mesh] OR "United Arab Emirates"[Mesh] OR "Yemen"[Mesh] OR "Pakistan"[Mesh] | -- | 136,850 |
| 7 | consumption[tiab] OR consuming[tiab] OR consume[tiab] OR consumes[tiab] OR intake*[tiab] OR food*[tiab] OR nutrition[tiab] OR diet*[tiab] OR source*[tiab] | -- | 2,161,793 |
| 6 | (((("Trans Fatty Acids"[Mesh]) OR (transfat*[tiab])) OR (trans-fat*[tiab])) OR (TFA[tiab])) OR ("partially hydrogenated"[tiab]) | -- | 7,155 |
| 5 | "partially hydrogenated"[tiab] | -- | 598 |
| 4 | TFA[tiab] | -- | 4,172 |
| 3 | trans-fat*[tiab] | -- | 2,987 |
| 2 | transfat*[tiab] | -- | 819 |
| 1 | "Trans Fatty Acids"[Mesh] | -- | 1,158 |
