# Supplementary material for: A Systematic Review of Trans Fat Reduction Initiatives in the Eastern Mediterranean Region
Source: Front Nutr. 2021 Nov 26;8:771492. doi: 10.3389/fnut.2021.771492 (PMC8662545; doi:10.3389/fnut.2021.771492)
Supplement: Supplementary file 2 [file Table_2.DOCX]

**Supplementary Table 2. Estimates of TFA Intakes in Countries of the EMR.**

| **Country** | **Reference** | **Year** | | **National or Regional** | **Method used** | **Study Population** | **Estimated TFA intake** |
| --- | --- | --- | --- | --- | --- | --- | --- |
| **EMR** | WHO global trans-fat elimination report 2019; Al Jawaldeh and Al Jawaldeh 2018; Al Jawaldeh and Al Jawaldeh 2018 (1-3) | -- | | -- | -- | -- | Mean intakes (% of EI):  1.9 |
| **Egypt** | Wang et al 2016 (4) | -- | | National | Dietary assessment | Entire population | Mean intakes (% of EI):  6.5 |
| **Iran** | Mozaffarian et al 2007 (5) | 2001-2003 | | National | 3 consecutive 24-hr dietary recalls | Urban and rural households from the 28 Iranian provinces;  N=7158 households, 35924 individuals | Mean intakes (g):  12.3  Mean intakes (% of EI):  4.2  Partially hydrogenated oils were commonly used for cooking in Iranian homes, with average per-person intake of 14 g/1000 kcal, representing 12.5% of all calories.  Per capita intake: 19 g/1000 kcal (with the additional intake of partially hydrogenated oils outside the home) (6) |
|  | Esmaeili et al 2014 (7) | 2014 | | National | 24-hr recall | Entire population | Mean intakes (g):  0.32 to 0.67 in different provinces |
|  | Amerzadeh et al 2020 (8) | 2018 | | National | Food consumption data | Entire population | Mean intakes (g):  1.5  Mean intakes (% of EI):  0.7 |
|  | Hosseini et al 2013 (9) | -- | | National | -- | Entire population | Mean intakes (g):  12.3 in 2007  1.42 in 2013 |
|  | Mirmiran et al 2019 (10)  Cohort study; Tehran Lipid and Glucose Study (TLGS) | 2006-2008 | | Regional; Tehran | Semi-quantitative FFQ | Children and adolescents aged 6-18 years;  N=424 | Mean intakes (% of EI) ± SD:  Boys: 2.26 ± 1.03  Girls: 2.20 ± 1.18 |
| **Jordan** | Alkurd 2011 (11) | 2006-2007 | | National | Based on the Jordanian Household Expenditures and Income Survey (JHEIS); questionnaire on expenditure on different foods | Households proportionally distributed across all governorates of Jordan;  N=12768 households | Daily per capita consumption of TFA: 1.25 g/day  TFA % share of TEE: 0.4% |
|  | Takruri and Alkurd 2014 (12)  Two-stage cluster stratified sampling | 2006-2007 and 2010 | | National | Based on the Jordanian Household Expenditures and Income Survey (JHEIS); questionnaire on expenditure on different foods | All governorates of Jordan;  N=13866 | *Daily intake of TFA (g) – 2006/2007 vs 2010:*  Amman: 0.91 vs 0.62  Balqa: 0.8 vs 0.63  Zarqa: 1.08 vs 1.03  Madaba: 1.13 vs 0.55  Irbid: 1.32 vs 0.82  Mafraq: 1.81 vs 0.7  Jarash: 1.03 vs 1.66  Ajloun: 1.89 vs 1.35  Karak: 1.16 vs 0.73  Tafilah: 0.95 vs 0.48  Ma’an: 1.97 vs 0.32  Aqaba: 0.89 vs 0.59  Entire country: 1.25 vs 0.72  *TFA % share of TEE – 2006/2007 vs 2010:*  Amman: 0.3 vs 0.2  Balqa: 0.2 vs 0.2  Zarqa: 0.3 vs 0.3  Madaba: 0.3 vs 0.1  Irbid: 0.4 vs 0.2  Mafraq: 0.6 vs 0.2  Jarash: 0.3 vs 0.4  Ajloun: 0.5 vs 0.4  Karak: 0.3 vs 0.2  Tafilah: 0.3 vs 0.1  Ma’an: 0.6 vs 0.1  Aqaba: 0.3 vs 0.2  Entire country: 0.4 vs 0.2 |
|  | Tayyem et al 2020 (13)  Cross-sectional, observational | 2017-2018 | | Regional; Amman | Semi-quantitative FFQ and 24-hr recall | Children and adolescents aged 6-18 years from selected schools;  N=136 | Mean intakes (g) ± SD:  FFQ: 0.8 ± 0.5; 24-hr recall: 1.3 ± 1.2 |
|  | Al-Jawaldeh and Al-Jawaldeh 2018 (2) | -- | | Regional; Amman | Dietary assessment | Adults | Mean intakes (% of EI):  Males: 0.5  Females 0.6 |
| **Lebanon** | Al-Jawaldeh and Al-Jawaldeh 2018 (2) | 2009-2011 | | National | Dietary assessment | Adults | Mean intakes (% of EI):  Males: 2.4  Females: 2.3 |
|  | Moghames et al 2016 (14)  Random cluster sampling design | 2011-2012 | | Regional; Beirut | Semi-quantitative FFQ and 24-hr recall | Children aged 5-10 years from public and private schools;  N=111 | Mean intakes (g) ± SD:  Boys: FFQ: 0.16 ± 0.14; 24-hr recall: 0.1 ± 0.11  Girls: FFQ: 0.15 ± 0.17; 24-hr recall: 0.1 ± 0.1  Total: FFQ: 0.16 ± 0.16; 24-hr recall: 0.1 ± 0.1 |
|  | Farhat et al 2016 (15)  Cross-sectional | 2009 and 2011 | | Regional | Diet history questionnaire (based on USA National Institute of Health (NIH)) and FFQ (quantitative web-based) | Convenience sample of Lebanese adults aged 19-70 years;  N=657 | Mean intakes (% of EI) ± SD:  Males: 2.4 ± 0.8  Females: 2.3 ± 0.8  *19-30 years:*  Males: 2.6 ± 0.9 and females: 2.4 ± 0.9  *31-50 years:*  Males: 2.1 ± 0.7 and females: 2.1 ± 0.7  *51-70 years:*  Males: 1.9 ± 0.6 and females: 1.8 ± 0.7  Mean intakes (g) ± SD by age groups:  *19-30 years:*  Males: 8.6 ± 5 and females: 2.4 ± 3.5  *31-50 years:*  Males: 7.2 ± 4.5 and females: 5.1 ± 2.8  *51-70 years:*  Males: 5.1 ± 3.5 and females: 3.4 ± 3.4 |
| **Morocco** | MOH and WHO 2020 (16) | -- | | National | Food consumption data | Entire population | Daily consumption data of the main sources of TFAs:   - Table oil: 47.84 g/person - Red meat: 81 g/person - Milk: 164 ml/person - Butter: 6.1 g/person - Margarine: 2.73 g/person - Cookies: 8.2 g/person - Yoghurts: 35.61 g/person   Daily intake (g) of TFAs:   - Table oil: 0.32 g - Butter: 0.21 g - Margarine: 0.2 g - Yoghurt: 0.02 g - Cookies: 0.01 g - Delicatessen: 0.0012 g   Mean intakes (% of EI):  0.34; this intake was calculated from the results of laboratory analyses of foods for which consumption data are available. |
| **Pakistan** | Wang et al 2016 (4) | -- | | National | Dietary assessment | Entire population | Mean intakes (% of EI):  5.7 |
| **Sudan** | Al-Jawaldeh and Al-Jawaldeh 2018 (2) | | 2006 | Regional; North and South | Dietary assessment | Female adults | Mean intakes (% of EI):  0.1 |
| **Tunisia** | Al-Jawaldeh and Al-Jawaldeh 2018; Al-Jawaldeh and Al-Jawaldeh 2018 (2, 3) | | 2005 | National | Dietary assessment | Adults | Mean intakes (% of EI):  Males: 0.1  Females: 0.1 |
| **UAE** | Ismail et al 2019 (17)  Cross-sectional | | 2017-2018 | Regional; Sharjah | 24-hr recall | Students from the University of Sharjah;  N=122 | Mean intakes (g) ± SD:  Males: 2.6 ± 1.6  Females: 1.9 ± 1.4  Mean intakes (% of EI) ± SD:  Males: 1.1 ± 0.6  Females: 1.0 ± 0.5 |
|  | Al-Jawaldeh and Al-Jawaldeh 2018 (2) | | 2014 | Regional; UAE university | Dietary assessment | University females | Mean intakes (% of EI):  1.7 |

Abbreviations: EI: energy intake; EMR: Eastern Mediterranean Region; FFQ: food frequency questionnaire; MOH: Ministry of Health; SD: standard deviation; TEE: total energy expenditure; TFA: trans fatty acid; UAE: United Arab Emirates; WHO: World Health Organization.

**References**

1. World Health Organization. Countdown to 2023: WHO report on global trans-fat elimination 2019 (2019). Geneva, Switzerland: World Health Organization. <https://apps.who.int/iris/bitstream/handle/10665/331300/9789241516440-eng.pdf?sequence=1&isAllowed=y>.

2. Al Jawaldeh A, Al-Jawaldeh H. Fat Intake Reduction Strategies among Children and Adults to Eliminate Obesity and Non-Communicable Diseases in the Eastern Mediterranean Region. Children-Basel (2018) 5:7. doi: 10.3390/children5070089.

3. Al Jawaldeh A, Al-Jawaldeh H. Scaling up obesity and NCD prevention in the Eastern Mediterranean Region through fat reduction intake strategies at population levels. Preprints (2018) doi: 10.20944/preprints201804.0218.v1.

4. Wang Q, Afshin A, Yakoob MY, Singh GM, Rehm CD, Khatibzadeh S, et al. Impact of nonoptimal intakes of saturated, polyunsaturated, and trans fat on global burdens of coronary heart disease. J Am Heart Assoc (2016) 5:1. 10.1161/JAHA.115.002891.

5. Mozaffarian D, Abdollahi M, Campos H, Houshiarrad A, Willett W. Consumption of trans fats and estimated effects on coronary heart disease in Iran. Eur J Clin Nutr (2007) 61:8. doi: 10.1038/sj.ejcn.1602608.

6. Nowrouzi F, Samimi B. The food balance sheet of Iran, 1989–2001. Agricultural Planning and Economic Research Institute: Tehran (2002)

7. Esmaeili M, Abdollahi M, et al. Effect of policymaking to reduce TFA intake in 6 provinces of Iran (2014). National Nutrition and Food Technology Research Institute

8. Amerzadeh M, Takian A. Reducing sugar, fat, and salt for prevention and control of noncommunicable diseases (NCDs) as an adopted health policy in Iran. Med J Islam Repub Iran (2020) 34:doi: 10.34171/mjiri.34.136.

9. Hosseini H, Abedi A, Abdollahi Z, Hajfaraji M, Mohammadi A. Fatty acid compositions of frequently consumed edible fat and oil in Iran with special emphasis on trans fatty acids (2013). Department of research of food technology, National Nutrition and Food Technology Research Institute, Shahid Beheshti University of Medical Sciences.

10. Mirmiran P, Ziadlou M, Karimi S, Hosseini-Esfahani F, Azizi F. The association of dietary patterns and adherence to WHO healthy diet with metabolic syndrome in children and adolescents: Tehran lipid and glucose study. BMC Public Health (2019) 19:1. doi: 10.1186/s12889-019-7779-9.

11. Alkurd RA. Estimated intakes of fats, cholesterol, fiber, sodium, calcium, potassium, and magnesium in Jordan. Aust J Basic Appl Sci (2011) 5:12.

12. Takruri HR, Alkurd RA. Intakes of fats, cholesterol, fiber and micronutrients as risk factors for cardiovascular disease in Jordan. Jordan J Biol Sci (2014) 7:2. doi: 10.12816/0008225.

13. Tayyem RF, Albataineh SR, Allehdan SS, Badran EF. Development and validation of a food frequency questionnaire for assessing nutrient intake during childhood in Jordan. Nutr Hosp (2020) 37:6. doi: 10.20960/nh.03079.

14. Moghames P, Hammami N, Hwalla N, Yazbeck N, Shoaib H, Nasreddine L, et al. Validity and reliability of a food frequency questionnaire to estimate dietary intake among Lebanese children. Nutr J (2016) 15:doi: 10.1186/s12937-015-0121-1.

15. Farhat AG, Jaalouk D, Moukarzel SR, Ayoub JJ. Consumption of trans fatty acid and omega 6 to omega 3 ratio in Lebanese adults. Nutr Food Sci (2016) 46:1. doi: 10.1108/NFS-07-2015-0089.

16. Ministry of Health-Morocco; World Health Organization. Reduction, replacement or elimination trans fatty acids in foods. 2020.

17. Ismail LC, Hashim M, Jarrar AH, Mohamad MN, Saleh ST, Jawish N, et al. Knowledge, attitude, and practice on salt and assessment of dietary salt and fat intake among university of sharjah students. Nutrients (2019) 11:5. doi: 10.3390/nu11050941.
