# Supplementary material for: A Systematic Review of Trans Fat Reduction Initiatives in the Eastern Mediterranean Region
Source: Front Nutr. 2021 Nov 26;8:771492. doi: 10.3389/fnut.2021.771492 (PMC8662545; doi:10.3389/fnut.2021.771492)
Supplement: Supplementary file 3 [file Table_3.DOCX]

**Supplementary Table 3. TFA Levels in Food and Meals.**

| **Country** | **Reference** | **Year** | **Method used** | **Food categories collected** | **TFA content in foods** |
| --- | --- | --- | --- | --- | --- |
| **Egypt** | Abdel-Moemin 2014 (1) | -- | Food samples were purchased from local markets in Cairo  Label content and chemical analysis | Six commercial dry soups | Label content of chemical analysis (TFA) – g/100 g:  - Commercial tomato soup with croutons: ND  - Commercial vegetable soup: ND  - Commercial dried lentil soup: ND  - Commercial chicken noodle soup: ND  - Commercial chicken soup with corn: ND  - Commercial cream of mushroom soup: ND  Study chemical analysis (TFA) – g/100 g:  - Commercial tomato soup with croutons: 0.268  - Commercial vegetable soup: 1.06  - Commercial dried lentil soup: 0.40  - Commercial chicken noodle soup: 0.25  - Commercial chicken soup with corn: 0.93  - Commercial cream of mushroom soup: 0.7  Reduction in content after pilot reformulation – g/100 g:  - Reformulated tomato soup with croutons: 0  - Reformulated vegetable soup: 0  - Reformulated dried lentil soup: 0  - Reformulated chicken noodle soup: 0  - Reformulated chicken soup with corn: not detected: ND  - Reformulated cream of mushroom soup: ND |
|  | El-Gawad et al 2015 (2) | -- | Food samples were obtained from local markets in Cairo  FAMEs and GC were used for analysis | Shortening (non-hydrogenated refined palm oil) and cocoa butter substitutes (hydrogenated pal kernel oil) | % TFA in the samples:  - Cocoa butter substitutes: 4.22 ± 0.007  - Shortening: 0.324 ± 0.006 |
|  | Sadek et al 2018 (3) | -- | Food samples of most frequently consumed fast foods; collected from restaurants in Qena city  FAMEs and GC were used for analysis | Grilled beef burger, grilled kofta, fried hotdog and fried chicken;  N=80 samples, 20 from each of the 4 | TFA in the samples (g/100 g) vs TFA (g) in 100 g of fat:  The fast foods samples contained TFA values ranging from 0.6-1.7 g/100g of food (3.5-11.3 g/100 g of fat):  - Grilled beef burger: 0.6 ± 0.7 g/100 g of food, 3.5 ± 4.1 g/100 g of fat  - Grilled kofta: 0.8 ± 0.8 g/100 g of food, 5.8 ± 5.4 g/100 g of fat  - Fried hotdog: 1.3 ± 0.7 g/100 g of food, 11.3 ± 9.3 g/100 g of fat  - Fried chicken: 1.7 ± 1.8 g/100 g of food, 10 ± 10.5 g/100 g of fat |
|  | Taher et al 2018 (4) | -- | Samples collected from dairy shops and street vendors in Giza and Qalyubiah governorates  FAMEs and GC were used for analysis | Fat-rich food products (cooking butter, margarine, ghee and shortenings)  N=120 samples, 30 from each of the 4 | % TFA in the samples:  Cooking butter: 0.58  Margarine: 0.19  Ghee: 1.01  Shortening: 0.23 |
|  | Ismail et al 2021 (5)  Based on the WHO protocol for measuring TFA in foods (6) | 2019 | Commonly consumed food sources in the Egyptian market, identified through literature review and market surveys, were considered  FAMEs and GC were used for analysis | 208 brands of commonly consumed foods: Fats and oils (n=42), milk and milk products (n=40), confectioneries (n=27), canned and frozen items (n=29), fast-food items (n=48) and sweets (n=22) | % TFA of total fats:  *Fats and oils:*  - Sunflower oil: 2.6  - Olive oil: 0.4  - Corn oil: 0.2  - Mixed oil: 2.9  - Yellow butter: 1.2  - Margarine: 0.9  - Coconut margarine; tehina: 0.8  - Mayonnaise: 2  *Milk and milk products:*  - Liquid milk (full cream): 2.1  - Powder milk (full cream): 1.4  - Powder milk (skimmed); coffee creamer: 1  - Roomy cheese: 2.2  - Mozzarella: 0.9  - Feta cheese: 1.5  - Creamy spread; processed cheese triangles: 1.7  - Processed cheese with cream cubes: 1.8  *Confectioneries:*  - Plain biscuits: 0.2  - Biscuits with chocolate: 2  - Plain cake: 0.5  - Chocolate cake: 2.6  - Crackers: 1.3  - Potato chips: 1.5  *Canned and frozen items:*  - Frozen pizza; frozen keba (meat and burghul): 2.2  - Frozen kafta (meat dish): 3.2  - Frozen mombar (sheep intestines with rice): 2.1  - Frozen nuggets: 1.3  - Luncheon, beef: 1.5  - Canned luncheon, beef: 0.4  - Canned tuna: 0.2  - Chicken cubes: 0.7  *Fast food items:*  - Hamburger: 1.3-1.9  - Shawarma; fried eggplants: 2.1  - Fried chicken: 2.7  - Falafel in new oil: 0.9  - Falafel in used oil: 4.8  - Fried potatoes in new oil: 0.5  - Fried potatoes in used oil: 4.7  - Sambosk cheese: 1.8  - Sambosk meat: 5.4  - Koshary (rice, lentils, pasta and onions): 0  - Kabab: 2.4-3.6  *Sweets:*  - Oriental sweets from specialized sweet shops:1.7  - Oriental sweets from bread bakeries: 3  - Zalabia (deeply fried flour batter): 0.1  - Doughnuts: 0.9  - Halawa: 0.7 |
| **Iran** | Bahrami et al 2003 (7) | -- | Different commercial brands of hydrogenated oils, currently available in Iran, were collected  HPLC was used for analysis | Hydrogenated oils | % TFA in the samples:  34.6 |
|  | Mozaffarian et al 2007 (8) | 2001-2003 | GC was used for analysis | Most consumed partially hydrogenated oils that were determined from the dietary data (24-h dietary recalls) | % TFA in the samples:  - Partially hydrogenated oil: 23.2-36.2  - Soybean oil: 0.9  - Sunflower oil: 4.7  - Olive oil: 0.4 |
|  | Asgary et al 2009 (9) | -- | Folch, FAMEs and GC were used for analysis | Four kinds of fast foods that are commonly consumed: - Sausage (red meat, powdered milk, soy protein, oil)  - Calbas (red meat, soy protein, oil)  - Hamburger (red meat, soy protein, oil)  - Pizza (sausage, calbas, cheese, red meat, tomato) | % TFA of total fats:  - Sausage: 26.2  - Calbas: 23.6  - Hamburger: 30.7  - Pizza: 28.2 |
|  | Asgary et al 2009 (10) | -- | Folch, FAMEs and GC were used for analysis | Oils marketed in Iran: PHVOs, cooking oils (non-hydrogenated vegetable oils) and frying oils | % TFA in the samples:  - Hydrogenated oils: 35.2%  - Cooking oils: 0.9%  - Frying oils: 2.6% |
|  | Butt and Sultan 2009 (11) | -- | -- | Low TFA Vanaspati ghee | % TFA in the samples:  7.7 |
|  | Nazari et al 2012 (12) | -- | Some common brands of the products were randomly chosen from different supermarkets  FAMEs and GC were used for analysis | Snack foods, dairy and bakery products; N=21 of each kind  -7 kinds of snack foods: cakes, cream biscuits, simple biscuits, cream chocolates, simple chocolates, potato chips and puffy  -8 kinds of dairy products: such as high-fat milks, low-fat milks, high-fat yogurts, low-fat yogurts, high-fat cheeses, low-fat cheeses, animal butters and plant butters | % TFA of total fats:  -Cakes: 36 ± 1  -Cream biscuits: 23 ± 13  -Simple biscuits: 24 ± 10  -Cream chocolates: 4 ± 32  -Simple chocolates: 8 ± 60  -Potato chips: 17 ± 38  -Puffy: 21 ± 94  % TFA of total fats:  -High-fat milk: 14.1 ± 10.9  -Low-fat milk: 9.2 ± 5.52  -High-fat yogurt: 2.9 ± 1.1  -Low-fat yogurt: 2.8 ± 0.7  -High-fat cheese: 3.3 ± 0.6  -Low-fat cheese: 1.3 ± 0.3  -Animal butter: 3.4 ± 0.7  -Margarine: 16.1 ± 7.88 |
|  | Hajimahmoodi et al 2013 (13) | -- | FAMEs and GC were used for analysis | 8 brands of liquid frying oil and 4 brands of solid oils | % TFA in the samples:  0.17-5.4 |
|  | Pasdar et al 2013 (14) | -- | Foods served in restaurants in Kermanshah  Folch and methylation methods were applied based on the guidelines of the AOCS; GC was used for the analysis | Meat products and kebabs; 14 types | % TFA in the samples:  - Loghmeh-kebab: 2.3  - Breaded shrimp: 0.1  - Chicken schnitzel: 0.13 |
|  | Teimouri et al 2014 (15) | -- | Samples were chosen by a random systematic method from producer list (obtained from health ministry) and were collected from different supermarkets in Iran  AOCO method and GC were used for analysis | Oils and food products; N=46 samples | % TFA in the samples:  - Margarine: 0-1.79  - Butter: 1.74  - Snack: 0.84 |
|  | Farmani and Gholitabar 2015 (16) | -- | Different brands were purchased from a local market in Amol  FAMEs and GC were used for analysis | Vanaspati fat; 10 brands | % TFA in the samples:  Range: 0.3-6.2; average: 3.1 |
|  | Abedi et al 2016 (17) | 2013 | Foods purchased from local supermarkets in Tehran  FAMEs and GC were used for analysis | 6 brands in 4 groups of edible oils and fats (partially hydrogenated vegetable oils, non-hydrogenated vegetable oils, frying oils and margarines) | % TFA in the samples:  Partially vegetable hydrogenated oils: 1.3-7.38  Non-hydrogenated vegetable oils: 0.45-1.2  Frying oils: 0.07-2  Margarines: 0.47-11.63 |
|  | Pasdar et al 2016 (18) | -- | Folch method was used for lipid extraction  AOCS method and GC were used for analysis | 13 of the most frequently consumed snacks (coffee mate, biscuits, cake, shortcake, donuts, bread tan, baklava, rice flour, chocolate, chips, snack, bamieh, zoolbia) | % TFA of total fats:  - Coffee mate: 13.94  - Biscuits: 12.86  - Cake: 6.95  - Shortcake: 3.38  - Donuts: 3.29  - Bread tan: 3.29  - Baklava: 2.5  - Rice flour: 1.89  - Chocolate: 1.24  - Chips: 0.61  - Snack: 0.52  - Bamieh: 0.2 |
|  | Chaharmahali et al 2018 (19) | -- | Samples were purchased from supermarkets  FAMEs and GC were used for analysis | 9 samples of edible oils and fat:  - Ladan sunflower oil  - Semen Sesame oil  - Familia virgin olive oil  - Bahar frying oil  - Pak unprocessed butter  - Shakelli unprocessed butter  - Shakelli Ghee  - Mahgol processed margarine  - Ladan Talaie partial hydrogenated oil | % TFA in the samples:  - Ladan sunflower oil: 1.07  - Semen sesame oil: 0.33  - Familia virgin olive oil: 0.39  - Bahar frying oil: 1.35  - Pak unprocessed butter: 3.78  - Shakelli unprocessed butter: 2.98  - Shakelli Ghee: 5.82  - Mahgol processed margarine: 4.76  - Ladan talaie partial hydrogenated oil: 0.61 |
|  | Saghafi et al 2018 (20) | 2014 | -- | Partially hydrogenated vegetable oils, non- hydrogenated vegetable oils, frying oils, margarines, shortenings, butters, and creams with different brands | % TFA in the samples:  - Shortening: 11.34  - Frying oil: 0.72 |
|  | Jamali et al 2019 (21) | 2016 | Samples were collected from the local market in Tehran  GC was used | Samples of cooking oil (N=5), frying oils (N=9) and table margarine (N=4) | % TFA in the samples:  - Cooking oil: 0.65  - Frying oil: 0.45  - Table margarine: 3.76 |
|  | Ghazavi et al 2020 (22)  Cross-sectional; descriptive, analytical | -- | Traditional sweets with traffic light labelling were randomly selected from confectionary shops in Isfahan  FAMEs and GC were used for analysis | 11 kinds of traditional sweets (9 samples of each); N=99  -Baghlava  -Bereshtuk sweet  -Date cookie  -Korki sweet  -Loz  -Chickpea sweet  -Qottab  -Yazd Brass sweet  -Kermanshah Brass bread  -Cookie  -Raisin cake brands | % TFAs of total fats vs. value of label:  -Baghlava: 0.46 ± 0.17 vs. 0  -Bereshtuk sweet: 2.50 ± 0.07 vs. 0.16  -Date cookie: 0.94 ± 0.10 vs. 0.1  -Korki sweet: 2.30 ± 0.90 vs. 0.16  -Loz: 0.04 ± 0.01 vs. 0.023  -Chickpea sweet: 2.30 ± 0.10 vs. 0  -Qottab: 1.40 ± 0.50 vs. 0.2  -Yazd Brass sweet: 0.85 ± 0.07 vs. 0.13  -Kermanshah Brass bread: 1.30 ± 0.20 vs. 0  -Cookie: 7.90 ± 0.07 vs. 2.7  -Raisin cake brands: 7.80 ± 1.60 vs. 0.2  Mean: 1.6 ± 0.3  % of non-compliance (TFAs content analyzed vs. value of label):  - 81.8% of the studied products showed a discrepancy in the TFAs in the values analyzed in laboratory. |
|  | Stender 2020 (23) | 2017 | Different products were purchased in Tehran  AOAC method was modified and used for analysis | Pre-packaged biscuits, cakes and wafers;  N=50 | 28 had values of TFA greater than 2% of total fat, ranging between 2 and 4.8%. The remaining 22 different products had TFA values below 2%.  Results showed that the amounts of industrial TFA in biscuits, cakes and wafers were minimal in Tehran in 2017. |
| **Jordan** | Mashai et al 2012 (24) | 2009 | Foods were purchased from superstores, fast-food restaurants, bakeries and sweets’ stores in Amman  FAMEs and GC were used for analysis | Local and imported commercial foods with diverse origin of fats | % TFAs of total fats:  - Bread and bakery: 2.46% (bread 2.14%; Arabic pastries 3.58%; Arabic cookies 1.74%; breakfast cereals 2.51%)  - Milk and dairy products: 3.8% (milk 3.89%; ice-cream 3.05%; Jameed 3.64%; Arabic cheese 4.8%; processed cheese 4.1%)  - Sausages and luncheon meats: 3.84% (sausage 4.01%; corned beef 5.54%; mortadella 3.33%)  - Fats and oils: 4.5% (vegetable oils 0.61%; margarine and butter 4.34%; mayonnaise 8.55%)  - Fast food: 4.16% (Instant soup 1.88%; shawarma 5.6%; falafel 4.08%; burgers 5.31%; pizza 4.43%; French fries 3.63%)  - Snacks: 3.93% (popcorn 20.38%; chips 1.47%; crackers 1.61%; sweet biscuits 2.82%)  - Baked sweets: 5.6% (baqlawa 4.67%; knafah bjibn 3.49%; doughnuts 20.76%; lazy cake 3.05%; vanilla cupcake 4.10%; chocolate cupcake 3.13%) |
|  | Al-Ismail et al 2021 (25)  Based on the recently developed WHO protocol for measuring TFA in foods (6) | -- | 14 of the selected food samples were prepared in the kitchen using the traditional preparation methods or according to the most used cookbooks in Jordan. The other 14 samples were purchased from local producers by taking 5 samples of each item from different producers and mixing them with equal amounts to prepare a representative composite sample  FAMEs and GC were used for analysis | A total of 22 traditional Jordanian sweet samples and 6 samples of appetizers | % TFA in the samples:  Ranged from ND to 0.4 |
|  | Unpublished data  Based on the recently developed WHO protocol for measuring TFA in foods (6) | -- | Samples of shortening and margarine were purchased from bakeries or local markets; sweets were collected from different sweet producers in Amman city  FAMEs and GC were used for analysis | 17 shortening samples, 12 samples of margarine and spreads, and 16 samples of traditional sweets | % TFA of total fats:  - Shortening: 0.16-0.89%, except for one sample which had 5.6% of TFA; with a mean of 0.29 ± 0.17%  - Margarine and spreads: 0.13-3.96% with a mean of 0.78 ± 1.15%  - Sweets: 0.14-0.66% with a mean of 0.38 ± 0.14% |
| **KSA** | Alfawaz 2004 (26) | -- | Foods obtained from local markets in Riyadh  GC was used for analysis | Different brands of vegetable oils used for cooking and frying, margarines, shortenings, and biscuits and cookies | % TFA in the samples:  - Vegetable oils were TFA-free  - Shortenings: 0.57-15.37  - Margarines: 0-4.53  - Biscuits and cookies: 0-23.12  - Extracted oil from French fried potatoes: 3.79-27.34  - Used frying oils from local restaurants and from a laboratory were also TFA-free |
|  | Bakeet et al 2013 (27) | -- | Products were purchased from retail supermarkets in Riyadh  FAMEs and GC were used for analysis | 4 brands of margarine  6 brands of shortenings | % TFA in the samples:  0.2-8.3% in margarines  0.9-20.6% in shortenings  Only one margarine and one shortening brand had total TFA less than 1%; all other samples had high total TFA content. |
|  | Kamel and Al Otaibi 2018 (28) | 2014-2016 | Food products were collected from local supermarkets in Al Ahsa to analyze the food labels information about TFA containing ingredients with different alternative terms | The food products items included: a) Bakery products: (n=74) such as biscuits, cakes, toast, wafers, pies, bread, crackers and samposa pastry; b) Dairy products: (n=67) included fermented milk, labenah, cream cheese spread, cheese slices, mozzarella, butter, cream and whipping cream; c) Other products such as dried soup, coffee mate, peanut butter and vegetable fat. | % of products declaring TF-containing ingredients in the ingredients list:  100% of pies and coffee mates, 66.66% of collected biscuits samples, 22.2% of vegetable fat and 12.5% of butter reported "hydrogenated oil" in their ingredients list.  50% of wafers listed "partially hydrogenated oil" as ingredient. Vegetable fat term was used with 38.89%, 66.67% and 33.33% in cake, bread and mozzarella products package, respectively.  33.33% of cakes and toasts using "margarine" term in their ingredients list. Generally, 1.42% of the samples used vegetable fat term and 35% used HO term.  Margarine and partially HO terms were used in 18% and 5%, respectively.  % of products declaring TF-content in nutrition facts:  - 11.11% of cakes  - 50% of biscuits and 50% of pies  - 100% of dressings and 100% of dried soups  - 0% of each of the following: toasts, wafers, breads, crackers, sambosa, mozarella, butter, whipping cream, coffemates, vegetable fat |
|  | Jradi et al 2020 (29) | 2016-2017 | 9 major food chains representing the largest retail brands, and 5 neighborhood grocery stores from the five regions of Riyadh—North, South, Central, West, and East were selected  Food was categorized, and data from labels and packages were collected | Categories were beverages, bakery products, canned foods, cereals and cereal products, confectionery, convenience foods, dairy products, fruits and vegetables, sauces, spreads and snack foods | Of the 1143 products that presented information on ingredients, 20% listed hydrogenated fat, 90% of which was fully hydrogenated, while 10% was partially hydrogenated. From the 228 products that had hydrogenated fat, 67.5% were imported, while 32.5% were locally produced.  The highest proportion of hydrogenated fat (56%) was found in confectionary products, followed by bakery products (52%). |
|  | *Information provided by the nutrition focal point* | 2016-2017 | Survey was conducted in the Riyadh market  Data from  labels and packages were collected | - Beverages (N=107)  - Bakery wares (N=115)  - Canned food (N=45)  - Cereals and cereal products (N=163)  - Confectionery (N=102)  - Convenience foods (N=73)  - Dairy products (N=133)  - Fruit and vegetables (N=68)  - Sauces and spreads (N=108)  - Snack foods (N=97) | % TFA in the samples*:*  - Beverages: 0, bakery wares: 0.4, canned food: 0, cereals and cereal products: 0, confectionery: 0.6, convenience foods: 0.1, dairy products: 0, fruits and vegetables: 0.2, sauces and spreads: 0, snack foods: 0.6.  The TFA levels were less than 1% in 10 major food groups, including beverages; bakery wares; canned food; cereals and cereal products; confectionery; convenience foods; dairy products; fruit and vegetables; sauces and spreads; and snack foods.  Proportion of products meeting SFDA for TFA declaration:  - Local products 42%  - Imported products 47%  - All products 46% |
| **Kuwait** | Sawaya et al 1998 (30) | 1995 | Kuwaiti composite dishes, commonly consumed, were chosen for this study; the dishes were prepared at the Ibn-Sina  Hospital kitchen of the Ministry of Public Health based on standardized recipes  GC was used for analysis | 22 Kuwaiti composite dishes | % TFA in the samples:  *Meat-based dishes:*  - Warag enab: 0.15  - Mahshi bil koosa: 0.35  - Gabbout: 0.2  - Tashreeb: 0.22  *Sweets:*  - Rangena: 0.2  *Cereal-based dishes:*  - Mashkoul: 0.04  - Khoubiz ragag: 0.03  - Khoubiz: 0.04  - Macaroni bil bashamel: 0.14  - Mashkoul bil bathengen: 0.05  TFA was not detected in the remaining dishes (3 meat-based dishes, 2 chicken-based dishes, 2 fish-based dishes, 4 sweets, 1 mixed salad). |
|  | Dashti et al 2003 (31) | -- | Kuwaiti composite dishes were chosen for this study  GC was used for analysis | 32 Kuwaiti composite dishes | % TFA in the samples:  *Kuba dishes:*  - Burgul kuba dish: 0.285  *Sandwiches:*  - Meat sandwiches: 0.118  *Dairy products:*  - Labneh: 0.227  - Halloumi: 0.348  TFA was not detected in the remaining dishes (7 fish-based dishes, 6 salads, 6 pastries, 4 sweets, chicken sandwich, 2 kuba dishes, 2 soups). |
|  | Al-Amiri et al 2020 (32) | -- | Foods were selected from “Kuwait Total Diet Study” data as the most commonly consumed local foods  AOAC method and GC were used for analysis | 21 foods commonly cooked and prepared at home, and 16 ready-made foods often purchased by locals rather than cooked at home | % TFA in the samples:  - Seafood: Marag Samak Maleh (Spanish mackerel broth): trace, Kabab Rubyan (Shrimps kebab): trace, Marag Rubyan (Shrimps broth): 0  - Soup: Shorbat Shaereya (Vermicelli soup): trace, Shorbat Dajaj (Chicken soup): 0.17, Shorbat Harees (Soup of peeled wheat with lamb): 0.24  - Cheese: Cream cheese spread, regular: 0.17, Triangle processed cheese: 0.28, “Al-Wafra” white cheese, low fat: 0.3  - Rice based: Aish Muhammar (Sweet rice): trace, Aish Muhammar (Sweet rice): trace, Aish Fagae ma Laham (Rice, truffle, lamb): 0.15, Aish Bajella ma Shbent (Rice, fava beans and dill): 0.38, Aish Tomat (Rice with tomato): trace, Kushari (Rice with macaroni and mung beans): trace  - Desserts: Gherayba (Shortbread cookie): 0.09, Knafah (Shredded phyllo dough in sugar syrup): 1.53, Baklawa (Phyllo pastry with nuts, Baklava): 1.5, Baklawa (Phyllo pastry with nuts, Baklava): 0.07, Samsamia (Sesame bar trace, Khabeesa (Cooked semolina with oil and sugar): trace, Darabeel (Rolled dough stuffed with sugar): 0.44, Zalabia (Jalebi): 0.4, Aseeda (Cooked wheat flour with sugar): trace, Halwa (Sweet wheat starch): 0.15, Baksam: 0.68  - Seed based: Heso: 0.2, Harda: trace  - Meat Based: Madhrouba: 0.39, Arayes: 4.78, Hameesat Kabdah: 0.28, Keema: 1.5  - Sandwich, cheeseburger: 2.33, chicken burger: 1.37  - Vegetable based: Hameesat Muchroom: trace, Hameesat Fagae: trace, Marag Hawa: trace |
|  | *Information provided by the nutrition focal point* | 2020 |  | Locally produced and imported food items that are commonly consumed by the public:  - Oils and Ghee: olive, sunflower, palm, corn oil  - Margarine  - Dairy: Milk- full cream/ long life, ice cream  - Pastry: Croissants, frozen pizza, puff pastry products  - Desserts and sweets: chocolates, biscuits, cupcakes, traditional sweets (nut and date-filled maamoul)  - Others: Peanut butter, popcorn (microwave prepared), readymade soup, karak tea (traditional), coffee whitener, readymade cake flour | Samples with % TFA below the detection limit:  - Full cream long life milk, ice cream vanilla, full cream fresh milk, sunflower oil, corn oil, olive oil, coconut oil, margarine, cake, biscuits, maamoul, crepe, chocolate, puff pastry, pizza, sweet bakasam, mini feta cheese patee, chicken noodle soup, instant coffee with creamer, microwavable popcorn.  Samples with detected %TFA:  - Cake vanilla mix: 0.14%  - Karak cardamom: 0.125% |
| **Lebanon** | Saade-Thesis 2007 (33) | 2005-2006 | Most frequently sold items and brand names, as indicated by supermarket and minimarket holders, were collected  FAMEs, according to AOAC procedures, and GC were used for analysis | Bakery and snack products:  The samples were classified into 12 different categories as follows: Plain cakes (4); vanilla flavored cakes filled with cream (2); chocolate cake (1); plain biscuits (2); wholemeal biscuits (2); cream-filled biscuits (2); cream-filled chocolate coated biscuit (1); chocolate-coated biscuit (1); biscuits coated and filled with chocolate (2); croissants filled with chocolate (3); chocolate-filled wafers (2); wafers coated and filled with chocolate (2) | % TFA of total fats:  - Plain cakes: 4.821 ± 4.015  - Vanilla flavored cakes filled with cream: 5.416 ± 0.023  - Chocolate cake: 0.096 ± 0.013  - Croissants filled with chocolate: 6.284 ± 1.592  - Plain biscuits: 3.112 ± 3.311  - Wholemeal biscuits: 0.185 ± 0.178  - Cream-filled biscuits: 4.570 ± 4.838  - Chocolate coated biscuit filled with cream: 0.353 ± 0.008  - Chocolate coated biscuit: 19.507 ± 0.158  - Chocolate coated biscuit filled with chocolate: 15.618 ± 7.676  - Chocolate filled wafers: 5.484 ± 2.321  - Wafers coated and filled with chocolate: 20.853 ± 1.526  The proportion of TFA ranged from 0.096 to 6.284% in bakery products and from 0.185 to 20.853% in snack products with the mean for snacks being higher than for bakery products (8.85% vs. 4.91%). |
|  | Nasreddine et al 2014 (34) | -- | -- | Biscuits  Bakery products  Snacks | % TFA in the samples:  For biscuits, 1/3^rd^ of the samples exceeded 5% of TFA (0.2–19.5%).  Bakery products (mean: 4.91 ± 3.11%, range: 0.10–6.28%) as well as snacks (mean: 8.85 ± 8.57%, range: 0.19–20.85%) |
|  | Saadeh et al 2015 (35) | 2006 | Selected products in the Lebanese market were collected  FAMEs, according to AOAC procedures, and GC were used for analysis | 10 biscuits (including plain, whole meal, cream-filled and chocolate-coated), 7 cakes (including plain, chocolate and cream-filled), 4 wafers (including plain, chocolate-filled, chocolate-filled and coated with chocolate) and 3 croissants (including plain and chocolate-filled) | % TFA in the samples:  - Cakes: range 1.1–2.5, Mean ± SD 1.1 ± 0.7  - Biscuits: range 0.1–5.4, Mean ± SD 2.1 ± 2.1  - Croissants: range 1.9–2.8, Mean ± SD 1.9 ± 0.9  - Wafers: range 1.2–8, Mean ± SD 4.6 ± 3.2  83.3% of the products contained more than 2 g TFA/100 g fat. |
|  | Hoteit et al, unpublished data  Based on the recently developed WHO protocol for measuring TFA in foods (6) | 2021 | Traditional dishes, Arabic sweets and most frequently consumed market foods  FAMEs, according to AOAC procedures, and GC were used for analysis | 30 types of traditional dishes, 35 types of Arabic sweets, 46 market foods (bread, biscuits, breakfast cereals, cakes, chocolate, chocolate wafers, coffee, croissant, nuts and seeds, doughnuts, Halawa, Chips, Tahina and canned tuna) and 34 vegetable oils and margarines | % TFA in 100 g of the samples:  - Traditional dishes: range 0.1-1.98, Mean 0.9  - Arabic sweets: range <0.1-1.45, Mean 0.6  - Margarines: range <0.1-11.8, Mean 2.4  - Biscuits, doughnuts, cake: range <0.1-2.6, Mean 0.5  - Cereals and breads group: range <0.1-0.7, Mean 0.3  - Tuna: range 0.3-0.6, Mean 0.45  - Chocolate and chocolate wafers: range <0.1-6.5, Mean 1.3  - Chips, nuts and seeds: range <0.1-0.3, Mean 0.3  - Tahina and Halawa: range 0.1-1.3, Mean 0.6  Only 7% of the products exceeded 2 g TFA/100 g fat. |
| **Morocco** | MOH and WHO 2020 (36) | 2020 | GC was used for analysis | Several food products | % TFA of total fats:   - Shortenings: 42.16 - Partially hydrogenated oils: 4.06 - Margarine: 3.67 - Butter: 3.12 - Table oil: 0.67 - Creams: 0.48 - Frying oil: 0.43 - Chips: 0.27 - Breakfast cereals: 0.16 - Cookies: 0.07 - Yoghurts: 0.06 - Delicatessen: 0.03 - Viennese pastry: 0.01 |
|  | Ministry of Health, unpublished (37, 38) | -- | -- | Margarines, fast foods and traditional foods | % TFA in the samples:  Margarine: 9.1–21.7  Fast foods: (average: 1.6 ± 1.1, range: 0.75–2.66)  Traditional foods: (average: 2.1 ± 1.9, range: 0.29–6.3) |
| **Pakistan** | Bhanger and Anwar 2004 (39); Butt and Sultan 2009 (11); Tarar et al 2020 (40) | 2004 | FAMEs and GC were used for analysis | 34 vanaspati (vegetable ghee)  11 shortenings  11 margarines | % TFA in the samples:  - Vanaspati: 14.2-34.3  - Shortenings: 7.3–31.7  - Hard-type margarines: 1.6–23.1  - Soft margarines: < 4.1 |
|  | Anwar et al 2006 (41); Butt and Sultan 2009 (11); Tarar et al 2020 (40) | 2004 | FAMEs and GC were used for analysis | Margarine, shortening and other food products | % TFA in the samples:  - Hard-type margarine: 1.6–23.1  - Soft-type margarine: < 4.1  - Shortening: 7.3–31.7  - Margarine: < 34.8  - Milk: 2.17–3.62  - Butter: 3.46–3.49  - Vanaspati: 14.43–14.46  - Tallow: 3.33–3.34  - Table margarine: 2.45–5  - Bakery margarine: 7.95–21.1  - Butter: 2.98–5  % TFA of total fats:  - Biscuits: 9.26–34.88 |
|  | Kandhro et al 2007 (42); Butt and Sultan 2009 (11) | -- | Samples were purchased from local supermarkets of Hyderabad; the choice of the brands was based on the highest consumption among those available in the market  FAMEs and GC were used for analysis | 10 margarine brands | % TFA in the samples:  Among samples tested only one contained a low level of TFA (2.2%) while the rest contained very high amounts of TFA (11.5–34.8%). |
|  | Kandhro et al 2008; Butt and Sultan 2009 (11, 43); Tarar et al 2020 (40) | -- | Biscuits purchased from local supermarkets in Jamshoro and Hyderabad  FAMEs and GC were used for analysis | 12 biscuits brands | % TFA of total fats:  9.3-34.9 |
|  | Butt and Sultan 2009 (11); Naz et al 2012 (44); Tarar et al 2020 (40) | 2012 | FAMEs and GC were used for analysis | Vegetable ghee and margarine (5 brands of each) | % TFA in the samples:  - Vegetable ghee: 5.36-33.03  - Margarine: 1.56-23.99 |
|  | Sherazi et al 2009 (45); Tarar et al 2020 (40) | 2009 | GC was used for analysis | Cooking oils; 14 samples | % TFA of total fats:  0.4-1.8 |
|  | Mahesar et al 2010 (46); Tarar et al 2020 (40) | 2010 | Foods purchased locally from supermarkets in Jamshoro  FAMEs and GC were used for analysis | 15 different cereal-based foods (crackers, cakes, mixed grains, corn chips, breakfast cereals) | % TFA in the samples:  2.5–16.3 |
|  | Sherazi et al 2010 (47); Tarar et al 2020 (40) | -- | Potato chips purchased from local supermarkets in Jamshoro and Hyderabad  FAMEs and GC were used for analysis | 12 potato chips brands | % TFA in the samples:  4.91-14.13 |
|  | Aftab et al 2012 (48); Tarar et al 2020 (40) | 2012 | Foods purchased from local supermarkets in Jamshoro  FAMEs and GC were used for analysis | 6 different chocolates and 5 different pastries | % TFA in the samples:  Chocolates: 4.56-8.49  Pastry: 3.92-10.17 |
|  | Karim et al 2014 (49); Tarar et al 2020 (40) | 2011-2012 | Foods purchased from different local retailers and a multinational fast food chain in Karachi  Attenuated total reflectance-FITR was used for analysis | French fries | % TFA in the samples:  0.11-24 |
|  | Shah et al 2016 (50); Tarar et al 2020 (40) | 2016 | Foods were collected from a local market as Faisalabad  Attenuated total reflectance-FITR was used for analysis | Traditional Pakistani breakfast and snack foods | % TFA of total fats:  Poori: 18.48%  Cake: 12.02%  Pratha: 11.01%  Doughnut: 38.69%  Puff pastry: 6.19% |
| **Tunisia** | El-Ati et al, unpublished data | 2018 | Brands of food products most commonly consumed by children aged 3-9 years in Greater Tunis were analyzed  AOAC procedures, and GC were used for analysis | 93 brands: 1 breakfast cereals brand; 54 cakes, sweets biscuits and pastries brands; 1 cheese brand; 13 chocolate and sugar confectionary brands; 5 composite foods brands; 1 ice-cream brand; 2 margarine brands; 4 processed foods brands; 8 savoury snacks brands; 5 spreads brands | TFA in the samples (g/100 g):  Breakfast cereals: 0  Cakes, sweet biscuits and pastries: 0-0.017  Cheese: 0.001  Chocolate and sugar confectionary: 0-0.006  Composite foods: 0-0.008  Ice-cream: 0  Margarine: 4.22-6.9  Processed foods: 0-0.003  Savoury snacks: 0.001-0.021  Spreads: 0-0.006 |
|  | Selmi et al 2019 (51) | 2017-2018 | 13 Tunisian fast food restaurants outlets were considered  Fat extraction of pre-dried food samples was carried out by soxhlet method | Chicken breast sandwich, Chicken fingers sandwich, Chawerma chicken sandwich, Tuna sandwich, Kaftaji sandwich, Salami and/or ham sandwich, Pie, Frying fats (margarines/oils) | % TFA of total fats:  - Chicken breast sandwich: 0.93  - Chicken fingers sandwich: 0.86  - Chawerma chicken sandwich: 0.71  - Tuna sandwich: 1.21  - Kaftaji sandwich: 0.81  - Salami and/or ham sandwich: 0.92  - Pie: 12.27  - Margarines: 1.01-9.9  - Frying oil: 1.29  - Commercial soybean oil: 0.18 |
|  | National Nutrition Institutes (38, 52) | -- | -- | Margarine and food items in general | % TFA in the samples:  One out of the two analyzed margarine brands exceeded 2% (1.4–9.8% of TF).  Relatively low TFAs levels were recorded with most food items ranging between 0.7% and 1.4% of total fat. |

Abbreviations: AOAC: Association of Official Agricultural Chemists; AOCS: American Oil Chemists' Society; FAMEs: fatty acid methyl esters; FITR: Fourier Transform Infrared Spectroscopy; GC: gas chromatography; HO: hydrogenated oil; HPLC: high-performance liquid chromatography; ND: not detected; KSA: Kingdom of Saudi Arabia; SFDA: Saudi Food and Drug Authority; TF: trans fat; TFA: trans fatty acid; WHO: World Health Organization.

**References**

1. Abdel-Moemin AR. Consumer satisfaction and nutrient profile of reformulated dry soups. Agricultura (Slovenia) (2014) 11:1/2.

2. Abd El-Gawad IA, Hamed EM, Zidan MA, Shain AA. Fatty acid composition and quality characteristic of some vegetable oils used in making commercial imitation cheese in Egypt. Journal of Nutrition and Food Sciences (2015) 5:4.

3. Sadek MAS, Daoud JR, Ahmed HY, Mosaad GM. Nutritive value and trans fatty acid content of fast foods in Qena city, Egypt. Nutr Food Sci (2018) 48:3. 10.1108/NFS-11-2017-0235.

4. Taher E, El-Essawy H, Saudi A, Aly S. Fatty Acid Profile of Some Fat Rich Foods with Special Reference to their Trans Fatty Acids Content. Int J Pharm Chem Sci (2018) 7:2.

5. Ismail G, Naga RAE, Zaki MES, Jabbour J, Al-Jawaldeh A. Analysis of fat content with special emphasis on trans isomers in frequently consumed food products in Egypt: the first steps in the trans fatty acid elimination roadmap. Nutrients (2021) 13:9. doi: 10.3390/nu13093087.

6. World Healh Organization. Protocol for measuring trans fatty acids in foods (2021). <https://www.who.int/docs/default-source/documents/replace-transfats/a-food-analysis-lab-protocol.pdf?sfvrsn=b27e4111_2>.

7. Bahrami G, Mirzaeei S, Kiani A, Atefi G. Assessment of profile of fatty acids and Trans fats in hydrogenated oils in Iran. J Kermanshah Univ Med Sci (2003) 7:1. doi: 10.22110/JKUMS.V7I1.860.

8. Mozaffarian D, Abdollahi M, Campos H, Houshiarrad A, Willett W. Consumption of trans fats and estimated effects on coronary heart disease in Iran. European journal of clinical nutrition (2007) 61:8.

9. Asgary S, Nazari B, Sarrafzadegan N, Parkhideh S, Saberi S, Esmaillzadeh A, et al. Evaluation of fatty acid content of some Iranian fast foods with emphasis on trans fatty acids. Asia Pac J Clin Nutr (2009) 18:2.

10. Asgary S, Nazari B, Sarrafzadegan N, Saberi S, Azadbakht L, Esmaillzadeh A. Fatty acid composition of commercially available Iranian edible oils. J Res Med Sci (2009) 14:4.

11. Butt MS, Sultan MT. Levels of Trans Fats in Diets Consumed in Developing Economies. J AOAC Int (2009) 92:5.

12. Nazari B, Asgary S, Azadbakht L. Fatty acid analysis of Iranian junk food, dairy, and bakery products: Special attention to trans-fats. Journal of research in medical sciences: the official journal of Isfahan University of Medical Sciences (2012) 17:10.

13. Hajimahmoodi M, Arami S, Nosrati M, Moghaddam G, Sadeghi N, Oveisi MR, et al. Trans Fatty Acid Content of Iranian Edible Oils. Food and Nutrition Sciences (2013) 4:11.

14. Pasdar Y, Bahrami G, Karvand F, Khodadoost M, Rezaei M, Niazi S. Determining the fatty acid content of the most common meat products in Kermanshah, Iran. Jorjani Biomedicine Journal (2013) 1:1.

15. Teimouri M, Najafi M, Eskandarion MR, Parto F. Assessment of Trans Fatty Acids content in Iranian food products. Innovative Food Technologies (2014) 2:1.

16. Farmani J, Gholitabar A. Characterization of Vanaspati Fat Produced in Iran. J Am Oil Chem Soc (2015) 92:5. 10.1007/s11746-015-2641-4.

17. Abedi AS, Hosseini H, Mohammadi A, Abdollahi Z, Hajifaraji M, Khaneghah AM. Fatty Acid (FA) Compositions and trans Content of Frequently Consumed Edible Oils and Fats from Iran' Market. Current Nutrition & Food Science (2016) 12:1. 10.2174/1573401311666150930215645.

18. Pasdar Y, Bahrami G, Alghasi S, Darbandi M, Hemati Azandaryani A, Bahrami S, et al. Trans Fatty Acids Contents among Selected Foods in Western Iran. International Journal of Health and Life Sciences (2016) 2:1.

19. Chaharmahali NV, Asadollahi S, Hosseini E. Measurement and comparison of trans fatty acids amount in some of the vegetable oils, frying oils and animal and vegetable fats in Iran. Int J Bio-Inorg Hybr Nanomater (2018) 7:1.

20. Saghafi Z, Zargaraan A, Tabibiazar M, Hosseini H. Is trans fatty acid still an issue for policy makers in Iran? A Technical Report. Nutrition and Food Sciences Research (2018) 5:2.

21. Jamali A, Moslemi M, Akramzadeh N, Khaneghah AM, Dadgarnejad M. Safety factors of oils marketed in Iran and applicable strategies in control of food derived cardiovascular diseases. Curr Nutr Food Sci (2020) 16:4. 10.2174/1573401315666181204110604.

22. Ghazavi N, Rahimi E, Esfandiari Z, Shakerian A. Accuracy of the amount of trans-fatty acids in traffic light labelling of traditional sweets distributed in isfahan, iran. Arya Atheroscler (2020) 16:2. 10.22122/arya.v16i2.2005.

23. Stender S. Trans fat in foods in Iran, South-Eastern Europe, Caucasia and Central Asia: a market basket investigation. Food Policy (2020) 96:doi: 0.1016/j.foodpol.2020.101877.

24. Mashai R, Ai-Ismail K, Al-Domi H, Al-Mousa T. Variability in trans fatty acid content of selected local and imported foods in Jordan. Riv Ital Sostanze Grasse (2012) 89:3.

25. Al-Ismail KM, Takruri HR, Tayyem RF, Al-Dabbas MM, Abdelrahim DN. Trans fatty acids content of sweets and appetisers traditionally consumed in Jordan. Riv Ital Sostanze Gr (2021) 98:1.

26. Alfawaz MA. Trans fatty acids in a selection of foods and oils available in Riyadh markets. Bulletin of Faculty of Agriculture, Cairo University (2004) 55:2.

27. Bakeet ZAN, Alobeidallah FMH, Shaista A. Fatty acid composition with special emphasis on unsaturated trans fatty acid content in margarines and shortenings marketed in Saudi Arabia. International Journal of Biosciences (IJB) (2013) 3:1.

28. Kamel S, Al Otaibi H. Trans-fats declaration, awareness and consumption in Saudi Arabia. Curr Res Nutr Food Sci (2018) 6:3. 10.12944/CRNFSJ.6.3.17.

29. Jradi H, AlMughthem A, Bawazir AA. Does the current Scope of nutrition labelling provided in the Saudi markets cope with the increasing trend of chronic disease? (2020) doi: 10.21203/rs.2.23201/v1.

30. Sawaya WN, Al-Awadhi F, Naeemi I, Al-Sayegh A, Ahmad N, Khalafawi MS. Dietary fat profiles of composite dishes of the Arabian Gulf country of Kuwait. J Food Compost Anal (1998) 11:3. doi: 10.1006/jfca.1997.0557.

31. Dashti B, Al-Awadi F, Sawaya W, Al-Otaibi J, Al-Sayegh A. Fatty acid profile and cholesterol content of 32 selected dishes in the state of Kuwait. Food Chem (2003) 80:3.

32. Al-Amiri HA, Ahmed N, Al-Sharrah T. Fatty acid profiles, cholesterol composition, and nutritional quality indices of 37 commonly consumed local foods in Kuwait in relation to cardiovascular health. medRxiv (2020) doi: 10.1101/2020.11.18.20233999.

33. Saade CN. (2007) Fatty acid composition, including trans fatty acids, of some local and regional bakery and snack products in the Lebanese market-by Carol Nakhle Saade. [Beirut, Lebanon]: American University of Beirut.

34. Nasreddine L, Naja F, Sibai A-M, Helou K, Adra N, Hwalla N. Trends in nutritional intakes and nutrition-related cardiovascular disease risk factors in Lebanon: the need for immediate action. Lebanese Medical Journal (2014) 103:1151.

35. Saadeh C, Toufeili I, Zuheir Habbal M, Nasreddine L. Fatty acid composition including trans-fatty acids in selected cereal-based baked snacks from Lebanon. J Food Compos Anal (2015) 41:10.1016/j.jfca.2015.01.014.

36. Ministry of Health-Morocco; World Health Organization. Reduction, replacement or elimination trans fatty acids in foods. 2020.

37. Ministry of Health-Morocco. Unpublished Report on the Fat Profile for Food Products. Rabat, Morocco: Ministry of Health; 2015.

38. Al Jawaldeh A, Al-Jawaldeh H. Fat Intake Reduction Strategies among Children and Adults to Eliminate Obesity and Non-Communicable Diseases in the Eastern Mediterranean Region. Children-Basel (2018) 5:7. doi: 10.3390/children5070089.

39. Bhanger MI, Anwar F. Fatty acid (FA) composition and contents of trans unsaturated FA in hydrogenated vegetable oils and blended fats from Pakistan. Journal of the American Oil Chemists' Society (2004) 81:2.

40. Tarar OM, Ahmed KM, Nishtar NA, Achakzai AB, Gulzar Y, Delles C, et al. Understanding the complexities of prevalence of trans fat and its control in food supply in Pakistan. The Journal of Clinical Hypertension (2020) 22:8.

41. Anwar F, Bhanger M, Iqbal A, Sultana B. Fatty acid composition of different margarines and butters from Pakistan with special emphasis on trans unsaturated contents. Journal of Food Quality (2006) 29:1.

42. Kandhro A, Sherazi S, Mahesar S, Bhanger M, Talpur MY, Rauf A. GC-MS quantification of fatty acid profile including trans FA in the locally manufactured margarines of Pakistan. Food Chemistry (2008) 109:1.

43. Kandhro A, Sherazi S, Mahesar S, Bhanger M, Talpur MY, Arain S. Monitoring of fat content, free fatty acid and fatty acid profile including trans fat in Pakistani biscuits. Journal of the American Oil Chemists' Society (2008) 85:11.

44. Naz R, Anjum FM, Rasool G, Nisar MA, Batool R, Saeed F. Total trans fat content in commercially available hydrogenated vegetable oils. Pak J Nutr (2012) 11:2.

45. Sherazi S, Kandhro A, Mahesar S, Bhanger M, Talpur MY, Arain S. Application of transmission FT-IR spectroscopy for the trans fat determination in the industrially processed edible oils. Food Chemistry (2009) 114:1.

46. Mahesar S, Kandhro AA, Cerretani L, Bendini A, Sherazi S, Bhanger M. Determination of total trans fat content in Pakistani cereal-based foods by SB-HATR FT-IR spectroscopy coupled with partial least square regression. Food chemistry (2010) 123:4.

47. Sherazi S, Kandhro AA, Mahesar S, Talpur MY, Latif Y. Variation in fatty acids composition including trans fat in different brands of potato chips by GC-MS. Pakistan Journal of Analytical & Environmental Chemistry (2010) 11:1.

48. Aftab A, Sherazi S, Rubina S, Razia S, Arfa Y. Consequence of fatty acids profile including trans fat in chocolate and pastry samples. Int Food Res J (2013) 20:2.

49. Karim Z, Khan KM, Ahmed S, Karim A. Assessment of trans fatty acid level in French fries from various fast food outlets in Karachi, Pakistan. Journal of the American Oil Chemists' Society (2014) 91:11.

50. Shah F, Rasool G, Sharif MK, Pasha I, Ahmad S, Sharif HR. Determination of trans fat in traditional Pakistani breakfast and snack foods. Int Food Res J (2016) 23:2.

51. Selmi S, Ghiloufi R, El-Ati J. Trans fatty acids content of selected popular Tunisian fast food. Journal of New Sciences (2019) 68:

52. National Nutrition Institutes. Unpublished Report Data on Food Consumption Pattern (2015). Tunis, Tunisia: National Nutrition Institutes.
