# Supplementary material for: A Systematic Review of Trans Fat Reduction Initiatives in the Eastern Mediterranean Region
Source: Front Nutr. 2021 Nov 26;8:771492. doi: 10.3389/fnut.2021.771492 (PMC8662545; doi:10.3389/fnut.2021.771492)
Supplement: Supplementary file 4 [file Table_4.DOCX]

**Supplementary Table 4. Knowledge, Attitudes and Behavior (KAB) towards TFA in countries of the EMR.**

| **Country** | **Reference** | **Year** | **National or Regional** | **Method used** | **Study Population** | **Findings** |
| --- | --- | --- | --- | --- | --- | --- |
| **Iran** | Dogani et al 2019 (1)  Cross-sectional, cluster sampling | 2016 | Regional; Fasa | Questionnaire | Adults;  N=990 households | - 31.5% of households use hydrogenated vegetable oils; 20.1% for cooking, and 11.4% for frying. |
|  | Borzouei et al 2010 (2)  Random selection of a representative sample | -- | Regional; Isfahan | Questionnaire | Females aged 18-28 years from Isfahan University of Medical Science; N=110 | - 7.3% used hydrogenated vegetable oils. |
| **Iraq** | MOH and WHO 2015 (3) | 2015 | National | STEPs Survey | Adults older than 18 years;  N=4059 | Oil or fat most often used for meal preparation in household: ghee 2.8%, margarine 0.1%. |
| **Jordan** | Al-Akash et al 2017 (4)  Cross-sectional, Convenience sampling | 2015-2016 | Regional; Amman | Questionnaire | Students at the University of Jordan;  N=417 | Frequency distribution (%) of students’ answers related to (knowledge; awareness):   - TFAs are contained in several food items like fast foods and snacks for example: Don’t know (27.1%); Know somehow (18.7%); Have fair knowledge (24.2%); Know very well (30%) - The maximum daily allowance of TFAs intake is 1% of the total energy requirements: Don’t know (53.2%); Know somehow (20.4%); Have fair knowledge (12.2%); Know very well (14.1%) - There are 2 types of TFAs according to their sources: natural and artificial: Don’t know (42.7%); Know somehow (24.7%); Have fair knowledge (16.8%); Know very well (15.8%) - Milk and its products and meat are considered the natural source for TFAs: Don’t know (35.5%); Know somehow (23.3%); Have fair knowledge (22.3%); Know very well (18.9) - The artificial source of TFAs is derived when vegetable oils are hydrogenated or exposed to heat: Don’t know (29.3%); Know somehow (22.5%); Have fair knowledge (23%); Know very well (25.2) - The artificial form of TFA is considered to be more harmful to health: Don’t know (22.3%); Know somehow (19.4%); Have fair knowledge (28.3%); Know very well (30) - TFA is contained in fast foods in variable amounts: Don’t know (20.4%); Know somehow (17%); Have fair knowledge (31.7%); Know very well (30.9%) - TFAs increase the risk for coronary artery diseases: Don’t know (15.3%); Know somehow (19.2%); Have fair knowledge (26.1%); Know very well (39.3%) - TFA increases the risk for diabetes mellitus: Don’t know (29.3%); Know somehow (18%); Have fair knowledge (27.1%); Know very well (25.7%) - TFA increases the risk for cancer: Don’t know (21.1%); Know somehow (22.1%); Have fair knowledge (27.3%); Know very well (29.5%) - My consumption of more than one TFA – containing food item means that I have exceeded my daily allowance of TFAs: Don’t know (24.7%); Know somehow (19.7%); Have fair knowledge (25.4%); Know very well (30.2%)   Descriptive statistics (%) of items containing artificial TFAs (%)-Correct=Contains; Incorrect- doesn’t contain:   - Vegetable ghee: correct (46.8%); incorrect (53.2%) - Butter: correct (61.4%); incorrect (38.6%) - Hard margarines: correct (42.9%); incorrect (57.1%) - Luncheon meat: correct (37.4%); incorrect (62.6%) - French fries: correct (77.5%); incorrect (22.5%) - Milk: correct (21.1%); incorrect (78.9%) - Fried chicken: correct (25.9%); incorrect (74.1%) - Arabic deserts (Baqlawa&Kenafa): correct (71.2%); incorrect (28.8%) - Chips: correct (81.3%); incorrect (18.7%) - Raw vegetables: correct (56.1%); incorrect (43.9%) - Dairy products: correct (16.5%); incorrect (83.5%) - Shawerma: correct (14.4%); incorrect (85.6%) - Falafel: correct (11.3%); incorrect (88.7%) - Doughnuts: correct (11.5%); incorrect (88.5%) - Fruits: correct (71.9%); incorrect (18.1%) - Croissant (Kerwasan): correct (54.7%); incorrect (45.3%) - Biscuits: correct (60.9%); incorrect (39.1%) - Avocado: correct (14.4%); incorrect (85.6%) - Nuts (walnut, almond, hazelnut, cashew): correct (15.8%); incorrect (84.2%) - Seeds (melon seeds, wheats, sissem): correct (15.3%); incorrect (84.7%) - Fish: correct (15.3%); incorrect (84.7%) - Liquefied vegetable oils: correct (16.1%); incorrect (83.9%) - Pizza: correct (69.1%); incorrect (30.9%) |
| **KSA** | MOH and WHO 2005 (5) | 2005 | National | STEPs Survey | Adolescents and adults aged 15 years and above | Type of oil and fat used:  Butter or ghee 2%  Margarine 0.9% |
|  | Kamel and Al Otaibi 2018 (6) | 2014-2016 | Regional; Al Ahsa | Dietary interview questionnaires | Adolescents and adults aged between 14 and 50 years | Awareness:   - Have you heard about Hydrogenated oil (HO)? Yes 35.1%, No 64.9% - Do you think that HO is unhealthy? Yes 4%, No 64.9%, I don’t know 31.1% - Are you interested in reading the food label? Yes 20.5%, No 79.5% - Do you buy HO to save money? Yes 48.3%, No 51.7%   Consumption of potential TFA-listing products sold in Saudi markets:   - Biscuits: 2-3 times per day 7.6%, once per day 7.9%, twice per week 41.7%, other 42.7% - Cake: 2-3 times per day 37.4%, once per day 15%, twice per week 16.9%, other 40.7% - Wafers: 2-3 times per day 24.5%, once per day 34.8%, twice per week 21.9%, other 18.9% - Pastries: 2-3 times per day 44.4%, once per day 12.9%, twice per week 19.9%, other 22.8% - Coffee mate: 2-3 times per day 23.5%, once per day 5%, twice per week 5%, other 66.6% |
| **Oman** | Manickavasagan et al 2014 (7) | -- | Regional; Muscat | Questionnaire  (based on the Omani Guide to Healthy Eating) | Students from 10 colleges | Awareness about the health effects of TFA:   - Good knowledge: around 25% - Little knowledge: around 30% - No knowledge: around 43% |
| **Pakistan** | Rashid et al 2020 (8) | -- | National | Situation analysis and gathering of evidence | -- | The research found extensive use of oils containing TFA among the Iranian public (up to 33% of fat in most oils). |
|  | Global Alliance for Improved Nutrition (GAIN) and SUN Business Network (SBN) (9) | 2020 | National | Secondary data | Households | % use of ghee and oil at the households:  45% of Pakistanis mostly use vanaspati ghee for cooking at homes; 40% mostly use packaged oil; 7% mostly use desi ghee. |
| **UAE** | MOHAP 2020 (10) | 2017-2018 | National | Population-based survey | Adults aged 18 years and above | Type of oil or fat most often used for meal preparation in the household:  Butter or ghee: Nationals: males 2.2%, females 2.8%; non-Nationals: males 2.1%, females 1.8%;  Margarine: Nationals: males 0%, females 0.1%; non-Nationals: males 0.2%, females 0.1% |

Abbreviations: EMR : Eastern Mediterranean Region; HO: hydrogenated oil; KAB: knowledge, attitudes and behavior; MOH: Ministry of Health; MOHAP: Ministry of Health and Prevention; TFA: trans fatty acid; UAE: United Arab Emirates; WHO: World Health Organization.

**References**

1. Dogani F, Najjari F, Naghizadeh MM, Dehghan A, Jeihooni AK, Askari A, et al. Analyzing Food Patterns and Preferences of Urban Population of Fasa (Phase I of Studying the Nutritional Healthy City in Fasa). Gazi Med J (2019) 30:4. doi: 10.12996/gmj.2019.91.

2. Borzouei A, Azadbakht L. Describing the dietary habits of Isfahan young girls: assessing the status of tea consumption, processed foods, fats and cooking methods. Health Syst Res (2010) 6:2.

3. Ministry of Health-Iraq; World Health Organization. Noncommunicable diseases risk factors STEPS survey Iraq 2015 (2015). <https://www.who.int/ncds/surveillance/steps/Iraq_2015_STEPS_Report.pdf>.

4. Al-Akash HY, AbuRuz ME, Arrah A. The University of Jordan Students' Concerns and Awareness about Trans-Fatty Acids. Adv Studies Biol (2017) 9:2. doi: 10.12988/ASB.2017.713.

5. Ministry of Health-Saudi Arabia; World Health Organization. WHO STEPwise approach to NCD surveillance: country-specific standard report (2005). <https://www.who.int/ncds/surveillance/steps/2005_SaudiArabia_STEPS_Report_EN.pdf>.

6. Kamel S, Al Otaibi H. Trans-fats declaration, awareness and consumption in Saudi Arabia. Curr Res Nutr Food Sci (2018) 6:3. doi: 10.12944/CRNFSJ.6.3.17.

7. Manickavasagan A, Al-Mahdouri AA, Al-Mufargi AMS, Al-Souti A, Al-Mezeini ASM, Essa MM. Healthy eating knowledge among college students in Muscat: A self reported survey. Pak J Nutr (2014) 13:7. doi: 10.3923/pjn.2014.397.403.

8. Rashid A, Amjad S, Nishtar MK, Nishtar NA. Trans-Fatty Acid (TFA) elimination in Pakistan: A situational analysis. J Pak Med Assoc (2020) 70 2):5.

9. Global Alliance for Improved Nutrition (GAIN); SUN Business Network (SBN). Mapping of Industrially-Produced Trans-fatty Acids (iTFA) in Pakistan (2020). Global Alliance for Improved Nutrition. <https://sunbusinessnetwork.org/mapping-industrially-produced-trans-fatty-acids-in-pakistan/>.

10. Ministry of Health and Prevention-UAE. UAE Nutritional Status Study. UAE: Statistic and Research Center; 2020.
