# Supplementary material for: A Systematic Review of Trans Fat Reduction Initiatives in the Eastern Mediterranean Region
Source: Front Nutr. 2021 Nov 26;8:771492. doi: 10.3389/fnut.2021.771492 (PMC8662545; doi:10.3389/fnut.2021.771492)
Supplement: Supplementary file 5 [file Table_5.DOC]

**Questionnaires sent to country nutrition focal points**

**TRANS FAT REDUCTION/ELIMINATION**

**COUNTRY QUESTIONNAIRE**

**Please provide references or relevant websites for all information provided below**

**Name of country:**

|  |
| --- |

**YOUR NAME, ORGANISATION and POSITION:**

|  |
| --- |

**YOUR CONTACT DETAILS:**

|  |
| --- |

**Please answer the following questions as accurately as possible or if you prefer, please attach the relevant documents and web links that provides answers to the questions.**

**What is the definition of Trans fat adopted in your country:**

| **Adopted definition with reference** |  |
| --- | --- |

**1. National trans fat reduction initiatives**

**1. Is there or has there previously been any program, policy or initiative designed to reduce trans fat in the food supply in your country?** *This includes initiatives led by government, industry, non-governmental organizations (NGOs) or any other agency, and may include any of the following: regulation, product reformulation, labelling, consumer awareness/ education, dietary target development, strategy development, monitoring/surveillance, research etc.*

| **YES** *(cont. ques 1)* | **NO** *(go to ques 2)* | **PLANNED** *(cont. ques 1)* |
| --- | --- | --- |

| **Name of initiative:** | | | **Timescale:** |
| --- | --- | --- | --- |
| **Is there a national target for trans fat intake?** | YES  NO  PLANNED |  | |
| **Who is the lead agency for the national trans fat reduction/elimination initiative?** | Government  NGO  Food Industry  Other, please specify: | | |
| **Is the initiative part of a broader health or nutrition program?** | YES (please indicate which program):  NO, the initiative is a trans fat-specific program | | |
| **Are there any NGOs or advocacy organisations active on trans fat reduction/elimination in the country?** | YES  NO | | |
| **Please provide reference for the above information** |  | | |

**2. Population trans fat intake**

**Has any work been done to measure the population’s trans fat intake in your country?**

| **YES** *(cont. ques 2)* | **NO** *(go to ques 3)* | **PLANNED** *(cont. ques 2)* |
| --- | --- | --- |

|  | **Year** | **Method used**  (E.g. dietary survey such as FFQ or 24 hour dietary recall, ……..) | **Estimated trans fat intake** (Total average, male average & female average) |
| --- | --- | --- | --- |
| **Please provide details on all the measurements of population trans fat intake in the country** (Insert additional rows if needed) |  |  |  |
|  |  |  |  |
|  |  |  |  |
|  |  |  |  |
| **Please provide reference for the above information** |  | | |

**3. Trans fat levels in foods and snacks**

**Has any work been done to determine the levels of trans fat in foods or snacks?**

| **YES** *(cont. ques 3)* | **NO** *(go to ques 4)* | **PLANNED** *(cont. ques 3)* |
| --- | --- | --- |

|  | **Year** | **Method used** (E.g. food analysis, survey, database, industry self-report) | **Food categories collected** | **Reduction in trans fat content demonstrated** |
| --- | --- | --- | --- | --- |
| **Does your country have food composition tables with data on trans fat** |  |  |  | YES. Please specify reference----------  NO |
| **Please provide details on all the measurements of trans fat levels in foods or snack categories in the country.** (Insert additional rows if needed) |  |  |  | YES. Please specify which foods: ….…...  NO |
|  |  |  |  | YES. Please specify which foods: ….…...     NO |
| **References** |  |  |  |  |
| **Do you have data on the major dietary contributors to trans fat intake in your country** | **Yea**r | **Method used** (survey, database) |  | **Results on main categories (% contribution)** |
|  |  |  |  |  |
|  |  |  |  |  |
| **References** |  | | | |

***4. Consumer knowledge, attitudes and behaviour***

**Has any work been done to determine the consumer’s knowledge, attitude and behaviour (KAB) in relation to trans fat?**

| **YES** *(cont. ques 4)* | **NO** *(go to ques 5)* | **PLANNED** *(cont. ques 4)* |
| --- | --- | --- |

|  | **Year** | **Method** (focus group, survey) | **Improvements in consumer KAB demonstrated** |
| --- | --- | --- | --- |
| **Please provide details on all the measurements of consumer knowledge, attitudes and behaviours in relation to trans fat in the country** (Insert additional rows if needed) |  | Survey  Focus group  Other | YES  NO |
|  |  | Survey  Focus group  Other | YES  NO |
| **References** |  | | |

**5. Implementation strategies**

**5A. ENGAGEMENT WITH INDUSTRY & REFORMULATION
Does your strategy include work with industry to achieve Trans fat reduction/elimination in foods?**

| **YES** *(cont. ques 5a)* | **NO** *(go to ques 5b)* | **PLANNED** *(cont. ques 5a)* |
| --- | --- | --- |

| **Name of initiative & year implemented** |  | | |
| --- | --- | --- | --- |
| **Agency/organization taking the lead to engage industry** | Government  NGO | Industry  Other, please specify: Bakeries and restaurants | |
| **Is the approach voluntary or mandatory?** | Voluntary  Mandatory for all food and snack categories (e.g. maximum trans fat content in foods and snacks or zero trans fat in all foods and snacks)  Mandatory for certain food and snack categories such as ……………. | | |
| **Approach to work with industry** | Meetings with companies  Voluntary commitments to trans fat reduction from companies  Cross-sectoral agreements to trans fat reduction (e.g. all snack manufacturers)  Targets for trans fat levels in foods and snacks  Taxation for high trans fat products  Other, please specify Workshops and Conferences for the industries and restaurants | | |
| **If trans fat reduction targets have been used, which food/snack categories have reformulation targets:** | Bakery products  Fast food  Restaurant frying oil  Margarines and spreads  Vegetable oils | | Biscuits and cakes  Salty snacks  Other, please specify …………….  Other, please specify ……………. |
| **Please indicate the targets that are set for the various food/snack categories (if available)** |  | |  |
| **References:** |  | |  |

**5B. CONSUMER EDUCATION/ BEHAVIOUR CHANGE
Does your strategy include activities to raise awareness/change behaviour on Trans fat?**

| **YES** *(cont. ques 5b)* | **NO** *(go to ques 5c)* | **PLANNED** *(cont. ques 5b)* |
| --- | --- | --- |

| **Name of initiative & year implemented** |  |  |
| --- | --- | --- |
| **Agency / organization taking the lead** | Government  NGO  Industry  Other, please specify: | Government  NGO  Industry  Other, please specify: |
| **Approach** | Social marketing (e.g. campaigns)  TV advertising  Events  Inclusion in FBDG  Other, please specify: | Social marketing (e.g. campaigns)  TV advertising  Events  Inclusion in FBDG  Other, please specify: |
| **References** |  |  |

**5C. FRONT OF PACK LABELLING
Has your country introduced front of pack labelling to indicate nutritional or trans fat content of foods/snacks?**

| **YES** *(cont. ques 5c)* | **NO** *(go to ques 5d)* | **PLANNED** *(cont. ques 5c)* |
| --- | --- | --- |

| **Name of initiative & year implemented** |  |
| --- | --- |
| **Agency / organization taking the lead** | Government  Industry  NGO  Other |
| **Is the approach voluntary or mandatory?** | Voluntary  Mandatory for all food categories  Mandatory for certain food categories such as ……………. |
| **Approach/detail of the label** (traffic light code, logo, % daily intake) | Traffic light code  Other logo or symbol  Warning labels  Percentage of daily intake  Other, please specify ……………. |
| **References** |  |

| **YES** *(cont. ques 5d)* | **NO** *(go to ques 6)* | **PLANNED** *(cont. ques 5d)* |
| --- | --- | --- |

**5D. WORK IN SPECIFIC SETTINGS
Does the strategy include work on Trans fat in particular settings such as schools, hospitals or workplaces?**

| **Name of initiative & year implemented** |  | |
| --- | --- | --- |
| **Setting** | School  Hospital | The workplace  Other, please specify ……………. |
| **Approach to work in particular settings** | Education  Procurement policy | Voluntary Guidelines  Other, please specify workshops |
| **References** |  |  |

**6. Evaluation**

**Has your country done any work to evaluate the overall effectiveness of the trans fat reduction strategy, in addition to monitoring trans fat intake, trans fat levels and consumer KAB? (For example process evaluation or cost effectiveness analysis)**

| **YES** *(cont. ques 6)* | **NO** *(end of survey)* | **PLANNED** *(cont. ques 6)* |
| --- | --- | --- |

**For each evaluation, please specify:**

(please copy and paste the table below as needed, one table per evaluation)

| **Year evaluated** |  |
| --- | --- |
| **Evaluation approach** |  |
| **Results  (Has it had an impact?)** |  |
| **Has a cost-effective analysis been undertaken?** | YES  NO  PLANNED |
| **Was the program cost-effective?** | YES  NO |
| **References** |  |

**7. Monitoring**

| **For the various trans fat reduction activities discussed in the questionnaire, please describe the monitoring system in your country:** |
| --- |
|  |

**8. Additional Information**

| **Did your country develop and regulation or reduction initiative pertinent to saturated fat?**  YES  NO  **If yes please elaborate:** |
| --- |

**Please attach any documents and website links relevant to the questions.**

**THANK YOU FOR COMPLETING THIS QUESTIONNAIRE. We value your time. Please send the completed questionnaire and relevant documents to Lara Nasreddine:** [**ln10@aub.edu.lb**](mailto:ln10@aub.edu.lb)

This questionnaire was developed based on the relevant literature, and particularly Downs et al (2013), Hyseni net al (2017) and Colon-Ramos et al (2013)
